# Macroporous chitosan/methoxypoly(ethylene glycol) based cryosponges with unique morphology for tissue engineering applications

**DOI:** 10.1038/s41598-021-82484-x

**Published:** 2021-02-04

**Authors:** Pradeep Kumar, Viness Pillay, Yahya E. Choonara

**Affiliations:** Wits Advanced Drug Delivery Platform Research Unit, Department of Pharmacy and Pharmacology, School of Therapeutic Sciences, Faculty of Health Sciences, University of the Witwatersrand, Johannesburg, 2193 South Africa

**Keywords:** Biotechnology, Biomaterials, Biomimetics, Regenerative medicine

## Abstract

Three-dimensional porous scaffolds are widely employed in tissue engineering and regenerative medicine for their ability to carry bioactives and cells; and for their platform properties to allow for bridging-the-gap within an injured tissue. This study describes the effect of various methoxypolyethylene glycol (mPEG) derivatives (mPEG (-OCH_3_ functionality), mPEG-aldehyde (mPEG-CHO) and mPEG-acetic acid (mPEG-COOH)) on the morphology and physical properties of chemically crosslinked, semi-interpenetrating polymer network (IPN), chitosan (CHT)/mPEG blend cryosponges. Physicochemical and molecular characterization revealed that the –CHO and –COOH functional groups in mPEG derivatives interacted with the –NH_2_ functionality of the chitosan chain. The distinguishing feature of the cryosponges was their unique morphological features such as fringe thread-, pebble-, curved quartz crystal-, crystal flower-; and canyon-like structures. The morphological data was well corroborated by the image processing data and physisorption curves corresponding to Type II isotherm with open hysteresis loops. Functionalization of mPEG had no evident influence on the macro-mechanical properties of the cryosponges but increased the matrix strength as determined by the rheomechanical analyses. The cryosponges were able to deliver bioactives (dexamethasone and curcumin) over 10 days, showed varied matrix degradation profiles, and supported neuronal cells on the matrix surface. In addition, in silico simulations confirmed the compatibility and molecular stability of the CHT/mPEG blend compositions. In conclusion, the study confirmed that significant morphological variations may be induced by minimal functionalization and crosslinking of biomaterials.

## Introduction

Three-dimensional porous scaffolds remain a critical component of the platform-based tissue engineering, reconstruction, and regenerative strategies^1^. These platforms are composed of natural and/or synthetic polymers, materials, and biomaterials capable of fulfilling certain essential requirements in terms of biocompatibility, biodegradability, encapsulation of bioactive components, easy formability, and controlled swelling^2^. In addition to the biomimetic, biocompatible, and biodegradability of these scaffolds; the morphological attributes such as porosity, size and shape of the pores, internal surface, roughness, and physical cues play an important role in the application of scaffolds for biomedical applications^3–5^. Another important consideration is the mechanical strength of the scaffolds to allow for easy handling and mechanocompatibility with the native tissue; and can be effectively determined as physical strength and deformation energy^6–8^.

Synthetic and natural polymers employed to prepare 3D scaffolds present their own challenges and advantages^9^. Synthetic polymers such as poly(lactic-co-glycolic acid) (PLGA), polycaprolactone (PCL), or poly(3-hydroxybutyrate-co-3-hydroxyvalerate) (PHBV) provide much required reproducibility, easy processability, and tuneable mechanical strength while they may lack biomimetic properties with reference to hydration and morphology^10,11^. Natural polymers such as chitosan, hyaluronic acid, and alginate may provide the extracellular matrix (ECM) mimicking properties but are comparatively less processable, lack mechanical strength, and are prone to microbial contamination^12^. Combining the above two classes of polymers to make a composite scaffold may address most of the concerns raised above and may even offer unique properties to the combination^13,14^. Natural-synthetic composite scaffolds may be formed as simple polymeric blends (using common solvent), interpolymeric complexes (using electrolytes), graft copolymers, or interpenetrating polymer networks (IPNs). Among these options, IPNs offer distinctive properties to the composite wherein one (semi-IPN) or both (full-IPN) the polymers are crosslinked in the presence of the other polymer. This way the inherent properties of the individual polymers (crosslinked or non-crosslinked) may be extracted as well as the network structure afforded by the IPN matrix may provide the required physical cues^15,16^.

Chitosan (CHT), a natural cationic glycosaminoglycan, is widely used for 3D scaffold preparation and ensuing tissue regeneration due to its unique properties including biocompatibility, biomimicking, biodegradability, bioactivity, and crosslinking ability as reviewed by several outstanding reports. In addition, this important biomaterial can be formulated into various platforms such as, but not limited to, sponges, cryogels, and dressings for drug delivery and tissue engineering applications^17–20^. Crosslinking agents for chitosan (glutaraldehyde, genipin, or tripolyphosphate) not only increase the mechanical properties of the scaffold but also prolong the retention time of the scaffold in vivo^21–24^. However, crosslinking may reduce the hydration profile of the chitosan scaffold requiring either the addition of a water soluble polymer or change in the method of crosslinking^25^. Polyethylene glycol (PEG), a synthetic polymer with a large range of molecular weight, is generally regarded as a biocompatible, non-toxic, and non-immunogenic biomaterial with high water solubility^26^. Given the above considerations, combinations of chitosan and PEG have been reported in literature either as PEG-*graft*-chitosan or as PEG-*blend*-chitosan for various biomedical applications such as controlled release, injectable systems, wound dressings, and tissue engineering, to name a few^27^.

In this study, chitosan was blended with various functionalized mPEG derivatives and crosslinked with glutaraldehyde under sub-zero temperature conditions to obtain semi-IPN cryosponges for potential neural tissue engineering applications. The effect of chemical functionalization of mPEG on the physicochemical, morphological, and mechanical properties of blend cryosponges was elucidated. A novel image processing protocol involving DiameterJ and ND plugins of ImageJ software was employed for analyses of the SEM micrographs in terms of porosity, pore wall thickness and roundedness of the pores. Additionally, the cryosponges were tested for matrix hydration and degradation parameters and for their drug release (dexamethasone and curcumin) ability. With minimal swelling, these scaffolds may afford specialized applications in neural tissue engineering. Physical testing involving matrix hydration and degradation was conducted in simulated cerebrospinal fluid for four weeks and the release of bioactives was observed for 10 days. This research reported mass loss as an indicator of degradation. Furthermore, preliminary cell studies were performed using a pheochromacytoma cell line (PC12) to assess the indicative biocompatibility of the cryosponges.

## Results and discussion

### Chitosan/mPEG-based cryosponges as biomaterial archetypes

**Figure 1:**
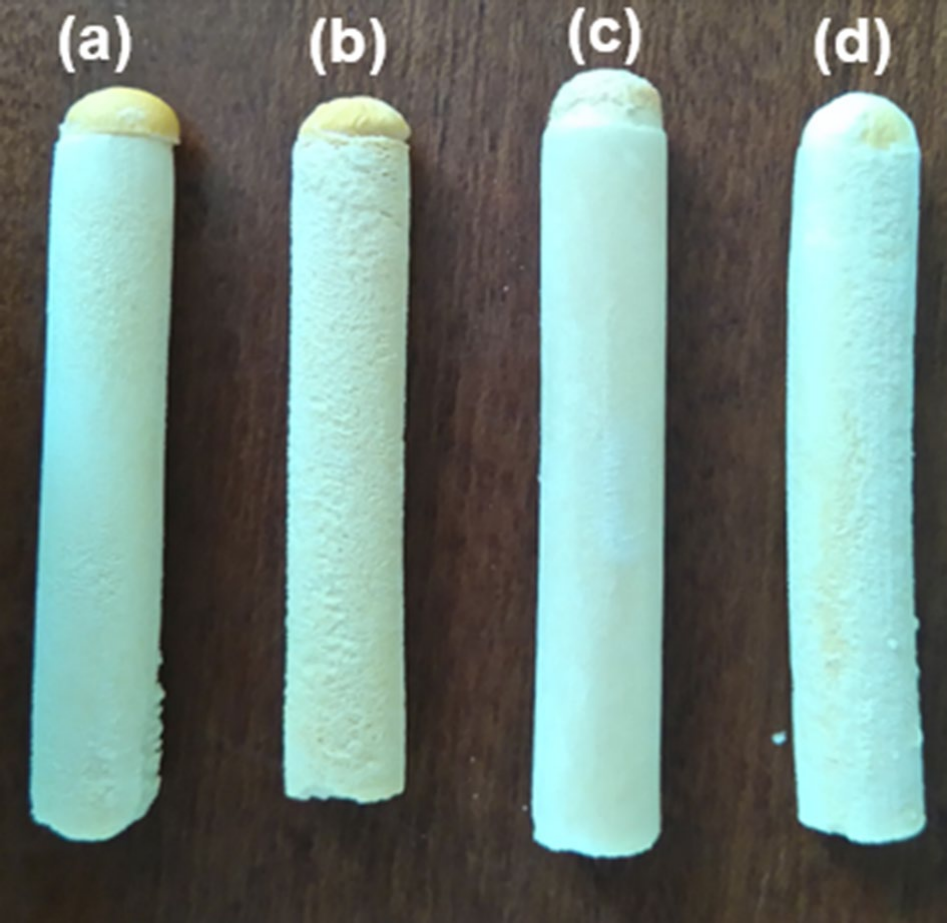
Photographs showing the physical form of (**a**) chitosan-alone; (**b**) chitosan/mPEG; (**c**) chitosan/mPEG-CHO; and (**d**) chitosan/mPEG-COOH cryosponges.

Chitosan/mPEG-based cryosponges were successfully prepared via the cryogelation technique (slow and controlled cryo-crosslinking). Perfectly cylindrical, uniform, macroporous cryosponges were obtained which maintained their cylindrical shape even after lyophilisation (Fig. 1). The cylindrical shape of the scaffolds may be directly applicable in conduit-based tissue engineering applications including spinal cord injury interventions. In addition, after rehydration, the scaffolds retained their original geometry and were robust to handle (using steel forceps) and cut (using razor blade) both in dried as well as hydrated states making them ideal candidates for commercial and clinical translation. This may further enable their application as custom-cut 3D scaffolds.

### Structural variations analysis using FTIR

FTIR analysis of the three mPEG derivatives showed perfectly overlapping, characteristic PEG spectra differing only with reference to wavenumber peaks corresponding to the functional group in the derivative. The strong bands at 2882 and 1341 cm^−1^ can be attributed to methoxy group (–OCH_3_) stretching and bending vibrations, respectively, and were present in all three spectra. The FTIR spectra of mPEG, mPEG-CHO, and mPEG-COOH showed characteristic –OH, –C=O stretch (aldehyde), and –C=O stretch (carboxylic acid) peaks at 3434.76 cm^−1^, 1737.13 cm^−1^ and 1752.01 cm^−1^, respectively. Additionally, a very broad –OH band was observed in for mPEG-COOH^28^. The absence of a –OH band in mPEG-CHO confirms the complete conversion of mPEG-OH to mPEG-CHO during synthesis^29^ (Supplementary material Figure S1 and Scheme S1).

The FTIR spectrum of plain chitosan showed characteristic bands at 3287 cm^−1^ (O–H axial stretching vibration, N–H extension vibration and polysaccharidic H-bonds overlap), 2871 cm^−1^ (axial stretching of C–H groups corresponding to –CH_2_ and –CH_3_ of pyranose ring), 1649 cm^−1^ (amide I C=O stretching band integrated with partial deacetylation), 1598 cm^−1^ (amide II N–H angular deformation + primary amine group and ammonium cation deformations + asymmetric C–N stretching band), 1419 cm^−1^ (confirmation of –NH_2_ groups), 1375 cm^−1^ (amide II –CH_3_ symmetrical angular deformation), 1318 cm^−1^ (C–N deformation related to amino groups), 1149 cm^−1^ (asymmetric C–O–C bridge stretching), and 1060/1024/990 cm^−1^ (C–O stretching vibrations characteristic of β(1 → 4) glycosidic bonds) (Supplementary material Figure S2)^30–32^.

The FTIR spectra of plain chitosan powder and the chitosan cryosponge confirmed that extensive vibrational transitions occurred with the formation of crosslinked cryosponge (Supplementary material Figure S2). Most of the vibrational bands corresponding to chitosan spectrum displayed a significant increase in the intensity after glutaraldehyde crosslinking. It was observed that four significant wavenumber changes occurred within chitosan to cryosponge transformation: 3300 cm^−1^ shifted to 3200 cm^−1^ and new (apparently) peaks appeared at 1636, 1545 and 1404 cm^−1^^33^. The sharpening and shifting of the broad 3300 cm^−1^ peak to 3200 cm^−1^ can be due to consumption of N–H stretching vibrations after reaction with –CHO of glutaraldehyde. Several researchers have ascribed the peak in the neighbourhood of 1630 cm^−1^ to vibrational stretching of C=N corresponding to the formation of a Schiff’s base with some studies referring to the simultaneous appearance of a C=C peak (corresponding to the amine catalysed aldol condensation and polymerization of glutaraldehyde) around 1550 cm^−1^^30,34,35^. However in the case of chitosan cryosponge reported in this study, the peak at 1636 cm^−1^ comparable to the relative transmittance with other peaks in the spectrum—no significant change in the relative intensity among the peaks (see % transmittance of 1649 cm^−1^ and 3300 cm^−1^ in chitosan vs that of 1636 cm^−1^ and 3200 cm^−1^ peaks in the cryosponge). Therefore the peak at 1636 cm^−1^ can be ascribed to a simple shift in the 1649 cm^−1^ waveband. This can be true due to the fact that the chitosan was not 100% deacetylated and the C=O bond in the amide (–NHCOCH) will remain intact.

With respect to the new strong peak at 1545 cm^−1^, it should be noted that in all previous studies, the C=N peak was the stronger peak or at the least both 1550 cm^−1^ and 1630 cm^−1^ peaks were of equal intensity. However in the case of chitosan cryosponge reported in this study, the peak at 1545 cm^−1^ appeared as the highest intensity peak and can be attributed to the formation of an imine bond between –NH_2_ and CHO. This can be supported by the fact that depending on the reaction conditions and the molecular structure of the reacting species, the imine band can appear anywhere between 1640 cm^−1^ and 1550 cm^−1^^36^. Souza and co-workers (2015) also reported C=N peak at 1536 cm^−1^ wherein chitosan was covalently crosslinked with squarate oxocarbon ions^37^. A further search of literature referring to C=N IR peak in organic compounds revealed that imine functionality can exist at or around 1545 cm^−1^^38^. Guan and co-workers also suggested a peak at 1551 cm^−1^ due to unreacted amino groups but the peak was very weak and hence not applicable to the current spectrum^39^.

The possibility of assigning the 1545 cm^−1^ peak to C=C can be argued to the fact that the imine/aldol conjugate forms in a 1:1 ratio and the peaks should be of equal intensities and hence not applicable in this case. The dominance of C=N peak (as compared to C=C or free amino groups) was further confirmed by the appearance of a strong Schiff’s base signature peak at 1404 cm^−1^^32^. Interestingly in the Handbook of Infrared and Raman Characteristic Frequencies of Organic Molecules (1991), it has been stated that alkyl-substituted ethylenes show a C=C stretching frequency in the neighbourhood of 1650 cm^−1^ which perfectly fits the alternative chitosan crosslinking mechanism of glutaraldehyde (imine formation + aldol condensation) proposed by Migneault et al.^40^. However, the intensity of 1636 cm^−1^ band was not significant to confirm this proposition.

In conclusion, the chitosan cryosponge was formed via a slow, low temperature, Schiff base formation crosslinking mechanism. The increase in the intensity of common peaks in the case of crosslinked chitosan scaffold as compared to chitosan powder can be attributed to the morphology of the scaffold as the functional groups are better exposed in an amorphous scaffold than the semi-crystalline powder when equivalent pressure is applied on the sample during FTIR analysis.

**Figure 2:**
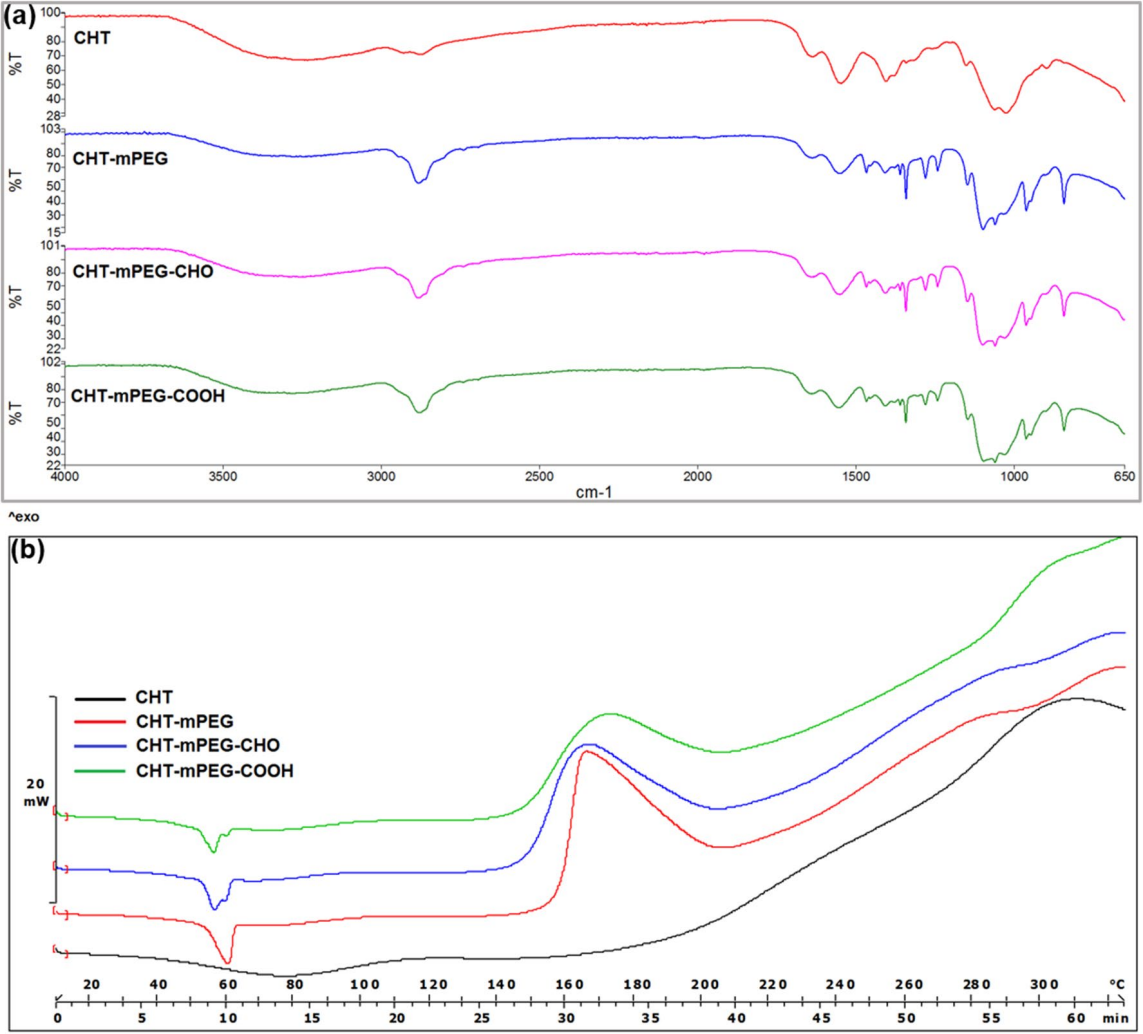
FTIR and DSC thermograms for CHT and CHT/mPEG cryosponges over various temperature ranges.

The FTIR spectra for various scaffolds are shown in Fig. 2. The CHT-mPEG spectra were composed of all the vibrational bands belonging to chitosan scaffold. However, the vibrational bands corresponding to PEG derivatives showed significant variations. One of the most noticeable changes involved the vibrational peak at 946 cm^−1^ corresponding to twisting and rocking –CH_2_– vibrations of PEG^41^. The original peak at 946 cm^−1^ involved a shoulder at 960 cm^−1^ while the CHT-mPEG spectra formed the peak at 961 cm^−1^ and a shoulder at 940 cm^−1^. This change can be attributed to the geometrical rearrangements and torsional strains experienced by the mPEG chain in close interaction and vicinity with the chitosan chain. It was hypothesized that the mPEG derivatives are capable of showing specific interactions with chitosan with mPEG forming non-bonding interactions; mPEG-CHO forming glutaraldehyde-type adduct, and mPEG-COOH forming an amide complex with the free –NH_2_ groups of chitosan.

This hypothesis was substantiated by the disappearance of characteristic –CHO (mPEG-CHO) and –COOH (mPEG-COOH) peaks from CHT-mPEG-CHO and CHT-mPEG-COOH spectra, respectively. To further confirm the disappearance/position of these peaks, deconvolution of the spectra was carried out between 1700 and 1800 cm^−1^. However, no peaks corresponding to 1737 and 1752 cm^−1^ were detected. When plotted in the overlay mode, the blend cryosponges’ spectra showed varied intensities at different wavenumbers with mPEG vibrations dominating the spectra except for 1636, 1545, and 1404 cm^−1^ peaks of chitosan scaffold. Among the three blend scaffolds, CHT-mPEG-CHO depicted peaks at 1551 and 1406 cm^−1^ confirming the involvement of mPEG-CHO in the formation of the C = N corresponding to the Schiff base^42^. Additionally, the CHT-mPEG-COOH spectrum showed a peak at 1639 cm^−1^ confirming the formation of an amide bond between mPEG-COOH and chitosan^43^. Since the mPEG derivatives were mixed with chitosan before adding the crosslinker, it appeared that mPEG chains interfered with the crosslinking action of glutaraldehyde decreasing the intensity of peaks representing native chitosan scaffold. This interference can be ascribed to the reduced availability or masking of chitosan functionalities due to geometrical conformational changes caused by non-bonding and bonding interaction with the PEG molecules as explained under the molecular modelling discussion.

### Thermoanalytical determination of structural transitions within CHT/mPEG cryosponges

Several studies had described the DSC scans of pure chitosan and PEG derivatives. Therefore, the DSC thermogram transitions observed in the cryosponges were described herein. The DSC analysis of the chitosan, mPEG derivatives and blend scaffolds was conducted over two temperature ranges (Fig. 2; Supplementary material Figures S3 and S4): 10–125 °C and 10–325 °C. The 10–125 °C DSC curves of plain chitosan powder and chitosan cryosponge provided broad endothermic peaks at 77.22 (− 189.76 J/g) and 78.25 (− 249.55 J/g), respectively, and can be assigned to the temperature of dehydration (Td). The increase in the Td for chitosan scaffold as compared to plain chitosan was due to reduced chain mobility of the rigid, crosslinked chitosan scaffold network (strengthened water-polymer interaction) requiring higher temperature and energy to release the bound water^44,45^. Additionally, the exothermic decomposition temperature peak for plain chitosan (295.33 °C) was increased to 312.17 °C in the case of chitosan scaffold further confirming the formation of a rigid, crosslinked network. The 10–125 °C curves of mPEG, mPEG-CHO and mPEG-COOH showed sharp melting endotherms at 62.76 (− 127.95 J/g), 59.50 (− 106.28 J/g), and 59.64 (− 114.63 J/g), respectively.

The melting endotherms in the case of mPEG-CHO and mPEG-COOH were comparatively broader at the top with the possibility of lower temperature shoulders. The 10–125 °C DSC curves of the blend scaffolds displayed a decrease in corresponding endotherm peaks compared to native mPEG polymers and chitosan scaffold as shown in Table 1. This observation can be attributed to a compatible blend formation via molecular interactions among the components of the blends as explained under FTIR and molecular modelling analysis^44,46^.

**Table 1:** Summary of thermal events within the DSC thermograms of CHT/mPEG cryosponges.

Formulation	Chitosan peaks (°C)		mPEG peaks (°C)		Enthalpy (J/g)
	Endo	Exo	Endo	Exo	
CHT (plain)	77.22	295.33	–	–	− 189.76
mPEG	–	–	62.76	252	− 127.95
mPEG-CHO	–	–	59.50	249.59	− 106.28
mPEG-COOH	–	–	59.64	290.97	− 114.63
CHT scaffold	78.25	312.17	–	–	− 249.55
CHT-mPEG	72.98	318.32	58.26	165.80	− 156.50
CHT-mPEG-CHO	71.95	320.65	54.90 (58.22)	167.50	− 140.81
CHT-mPEG-COOH	68.50	ND	55.56 (58.60)	176.88	− 136.00

Values in parentheses: higher temperature shoulder.

ND: not occurred under the tested temperature range.

Interestingly, CHT-mPEG-CHO and CHT-mPEG-COOH demonstrated clear lower temperature shoulders and newly formed higher temperature shoulders at 58.22 and 58.60 °C, respectively. The lower shoulders can be assigned to the formation of lamellae due to PEG chain extension or folding^47^. The new endothermic transitions at higher temperature shoulders may be caused by the formation of a mesophase—supermolecularly ordered, discrete phase—at the site of CHO–NH_2_ and COOH–NH_2_ interactions in CHT-mPEG-CHO and CHT-mPEG-COOH^48^. This discrete phase formation in the scaffolds may be responsible for the formation of the protruding structures (as shown under the SEM micrographs) and vice versa.

While the endothermic peaks of chitosan scaffold transitioned towards lower temperatures, the exothermic decomposition peak moved to higher temperatures (Table 1)—confirming the enhanced stability of the blends against the individual components. A decrease in both the endothermic peaks in the blends (corresponding to chitosan and mPEG derivatives) may be due to the plasticizing effect of mPEG on the chitosan indicating improved flexibility^49–51^ (Table 1). Another important thermal event within the blend scaffolds involved the notable transition of large exothermic peaks of mPEG, mPEG-CHO and mPEG-COOH at 252.15, 249.59 and 290.97 °C to 165.80, 167.50 and 176.88 °C, respectively. However, it should be noted that in DSC scans of plain polymers, the exothermic peaks started around 120 °C (mPEG and mPEG-CHO) and 150 °C forming broad thermal bands. The processing of mPEG polymer chains while forming the cryosponge and subsequent lyophilization streamlined the extension or folding architecture of PEG to a more consistent one leading to the formation of sharp well defined exothermic peaks starting around 140 °C—confirming the enhanced stability of mPEG chains in the cryosponges. It should further be noted that this mPEG exothermic peak overlapped with the broad chitosan endotherm peak (190 °C) starting at 140 °C. In conclusion, DSC substantiated the experimental observations made under FTIR, SEM and molecular modelling simulations.

### Morphological and image analyses of CHT/mPEG cryosponges

**Figure 3:**
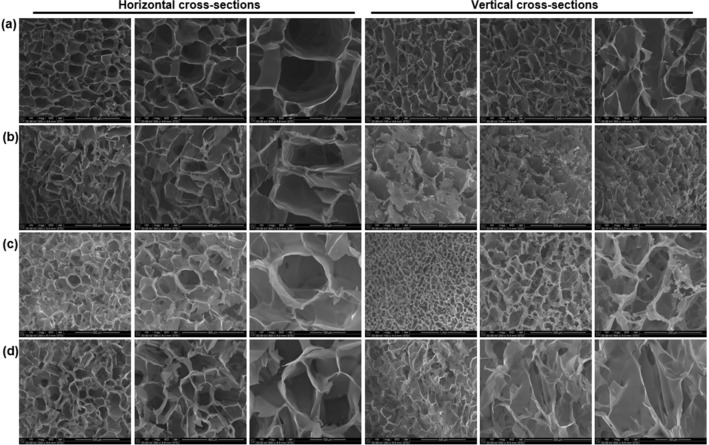
Scanning electron micrographs of (**a**) CHT; (**b**) CHT-mPEG; (**c**) CHT-mPEG-CHO; and (**d**) CHT-mPEG-COOH cryosponges representing horizontal cross-sections and vertical cross-sections.

**Figure 4:**
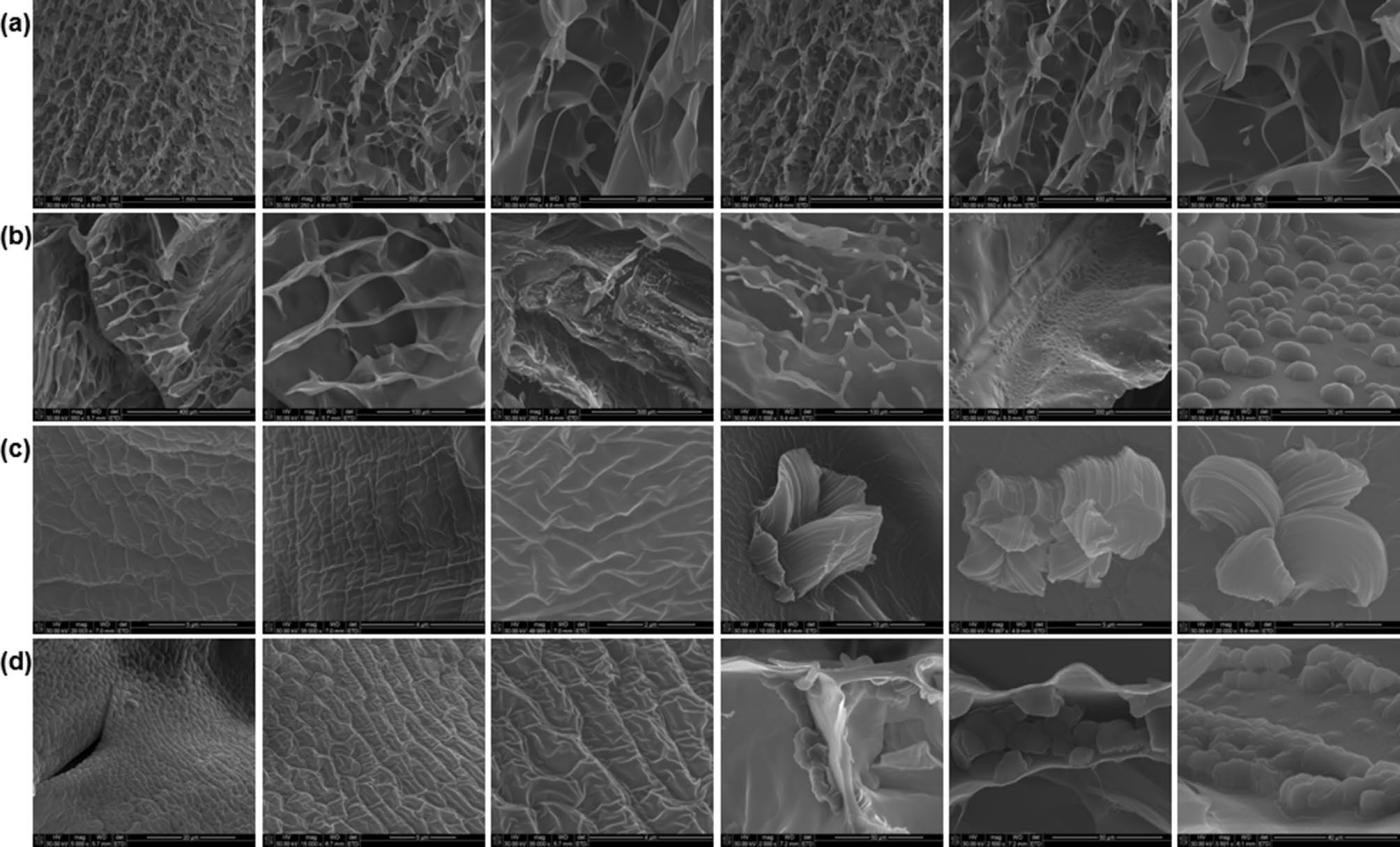
Scanning electron micrographs of (**a**) CHT; (**b**) CHT-mPEG; (**c**) CHT-mPEG-CHO; and (**d**) CHT-mPEG-COOH cryosponges representing surface characteristics and unique features of the porous network.

**Figure 5:**
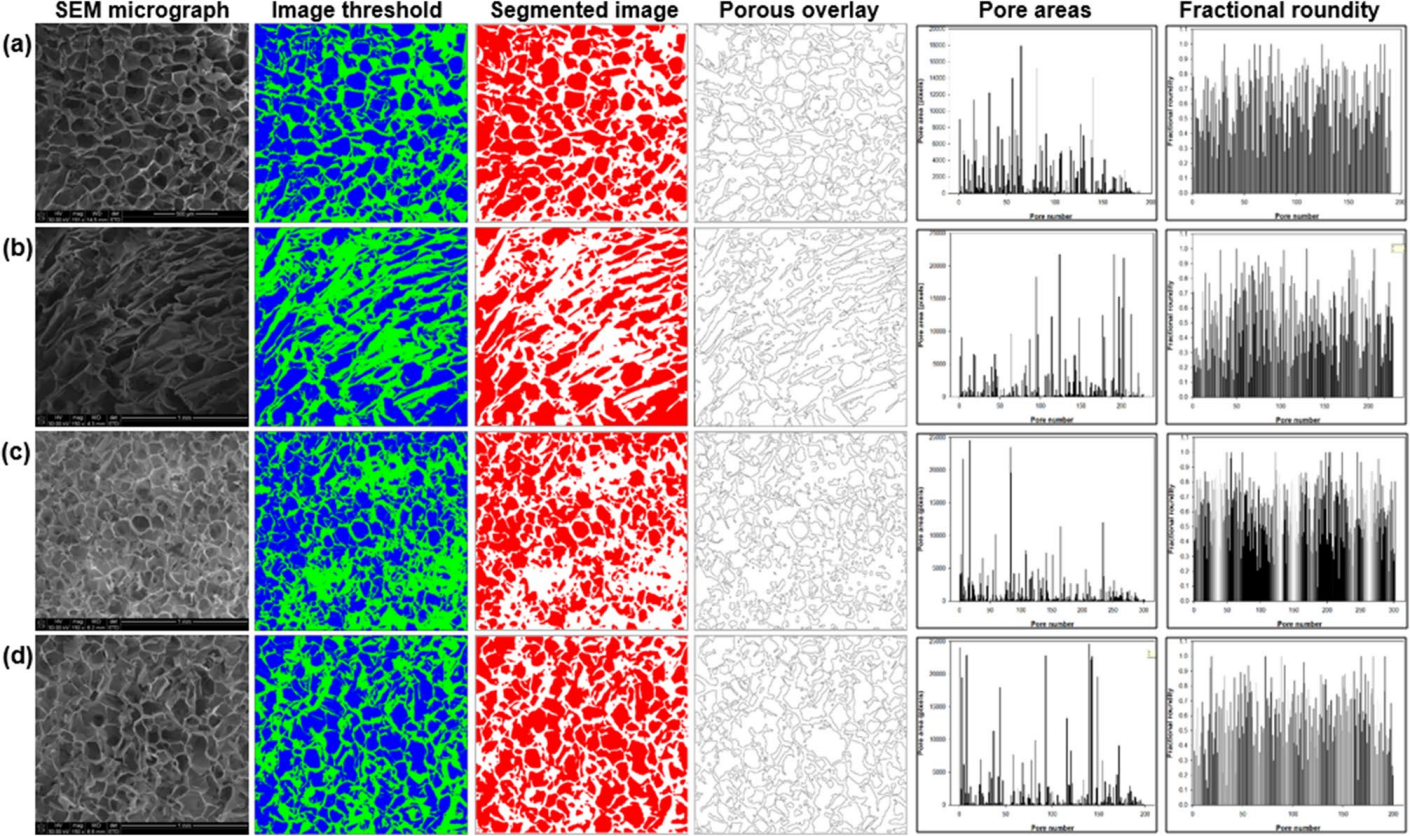
Image processing analysis of the porous architecture of (**a**) CHT; (**b**) CHT-mPEG; (**c**) CHT-mPEG-CHO; and (**d**) CHT-mPEG-COOH cryosponges obtained using ImageJ. Pore areas are presented as pixels (1 pixel = 1.66 µm).

The addition of PEG derivatives had a significant effect on the morphology of the CHT scaffold as shown in Figs. 3, 4, and 5. The scaffold cross-sections were scanned for horizontal and vertical pores. The surface of the pores was also scanned wherein some unique morphological features among the scaffolds were reported. The scaffold cross-sections were highly porous in nature and hence image processing was employed to obtain quantitative morphological data in terms of pore area, circularity of the pores and the distance of one pore from its four nearest neighbours (pore wall thickness). The brightness-and-contrast of the SEM images were adjusted to reach a threshold followed by segmentation of the images to achieve best possible differentiation between the pores and the solid surface. This is first such image processing involving ImageJ adjustment function along with two specialized ImageJ Plugins: ND (for pore size) and DiameterJ (for image segmentation and processing)^52,53^.

With the addition of PEG derivatives to chitosan, several interesting morphological changes with reference to the pore structure, pore surface and the presence of some unique structures within each scaffold were observed. Addition of mPEG to CHT transformed the fringe-like structures of CHT to fringe-with-bulb-like structures which appeared from the surface of the CHT-mPEG scaffold rather than from the edge of the pore in CHT. The plain surface of CHT scaffold appeared to cushioned together to form thin ridges in CHT-mPEG. The CHT-mPEG scaffold also marked the appearance of unique pebble-like structures on the scaffold surface. The surface further transformed into shallow, parallel ridges with the addition of mPEG-CHO to CHT making the surface rough. The pebble-like structures now changed to crystal-flower like structures with ridges in the petal. Finally, mPEG-COOH when added to CHT provided deep, elephant skin-like ridges thereby further increasing the roughness of the scaffold.

The unique, individually appearing, protruding structures obtained in CHT-mPEG and CHT-mPEG-CHO converted into steep-sided, canyon-like structures which grouped together within the porous architecture of the CHT-mPEG-COOH scaffold. The horizontal pores transformed as square → rectangular → round → irregular shape for CHT → CHT-mPEG → CHT-mPEG-CHO → CHT-mPEG-COOH, respectively. The salient features of the various scaffolds are presented in Tables 2 and 3. These morphology transformations can be attributed to the bonding and non-bonding interactions between chitosan and the PEG molecules. As explained further under molecular modelling simulations, addition of mPEG and its derivatives brought about geometrical changes in the polysaccharide chain due to destabilization of bending and torsional vibrations leading to significant morphological transformations. To the best of our knowledge, the unique structures obtained in this study had never been reported before. It was theorized that these protrusions appeared due to slow crosslinking reaction while forming the cryosponge giving sufficient time for the molecules to interact and form the scaffold. The appearance of the protrusions due to interaction-mediated mechanical strain can further be related to the appearance of mountains from plain land in response to mechanical strain experienced within the earth due to movement of tectonic plates.

**Table 2:** Porosity parameters for CHT/mPEG cryosponges computed using ND image processing.

Slice	Average cross-sectional pore area (µm^2^)	Pore area (%)	Roundity (%)
CHT	7508.70	58.681	64.6
CHT-mPEG	8271.21	49.117	62.0
CHT-mPEG-CHO	3986.79	49.262	70.3
CHT-mPEG-COOH	7273.42	59.518	64.8

**Table 3:** Qualitative, observational and comparative analysis of the CHT/mPEG cryosponges.

Scaffold architecture	Horizontal pores	Vertical pores	Characteristic structures
CHT	Square pores with round edges	Cuboidal, continuous, aligned pores	Fringe thread-like structures protruding from the edge of the vertical pore walls
CHT-mPEG	Rectangular, longitudinal, slit-like pores with defined edges	Layered pores with angular orientation appeared like flat sheets from top	Hemispherical, pebble-like structures on the surface of the scaffold
			Fringe thread-like structures with bulbs at the end protruding out from the base of the pores
			Parallel, thin-walled, flap-like ridges
CHT-mPEG-CHO	Round pores	Longitudinal, continuous, hybrid surfboard-shaped pores	Curved quartz crystal-like or crystal-flower-like structures protruding out from the surface/wall of the pores
			Rough shallow ridges on the surface of the scaffold
CHT-mPEG-COOH	Irregular shaped pores	Longitudinal, continuous, longboard-shaped pores	Grouped, congealed, steep-sided canyon-like structures protruding out from the surface of the pores
			Rough, deep, elephant-skin-type ridges on the surface of the pores

### Porositometric evaluation of the CHT/mPEGcryosponges

**Figure 6:**
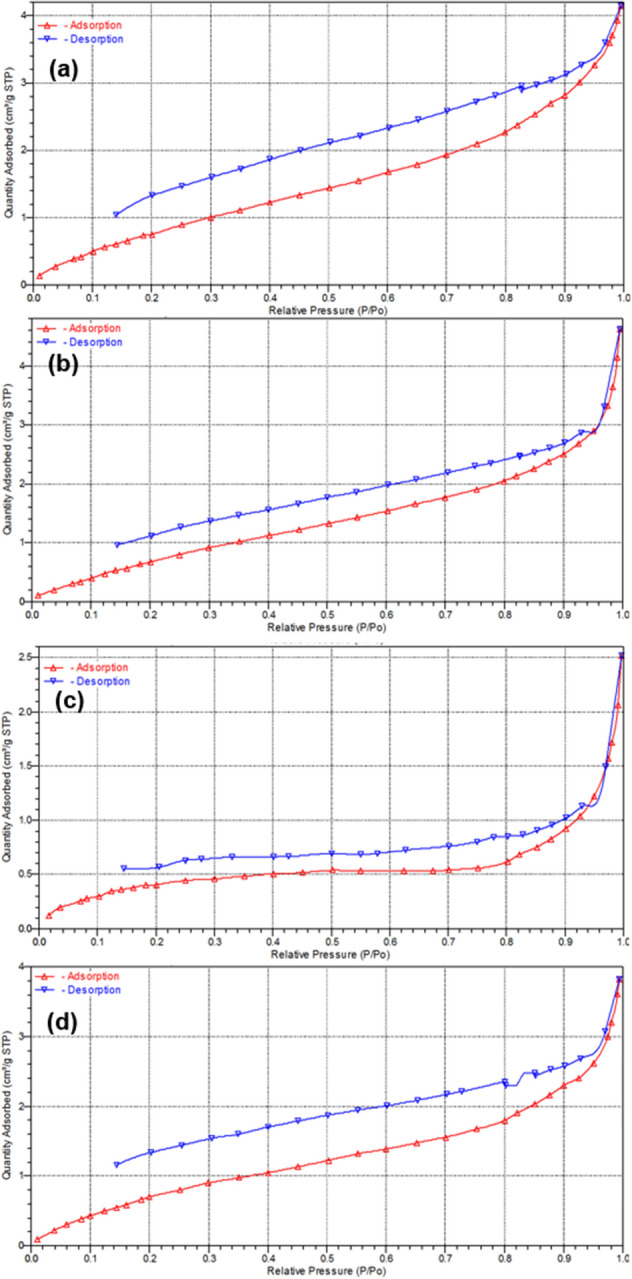
Linear isothermic plots of (**a**) CHT; (**b**) CHT-mPEG; (**c**) CHT-mPEG-CHO; and (**d**) CHT-mPEG-COOH.

To develop and analyse the adsorption–desorption isotherms of the cryosponges scaffolds, the porositometric analysis was carried out for CHT and was compared with that of CHT-mPEG blend scaffolds (Fig. 6; Table 4). For the interpretation of porosity profiles, IUPAC recommendations proposed by the Subcommittee on Reporting Gas Adsorption Data were referred to and the physisorption curves were compared with the isotherm types and hysteresis loops according to the IUPAC classification system^54,55^.

**Table 4:** Surface area and porosity characteristics of the various cryosponges.

Parameter	CHT-alone	CHT-mPEG	CHT-mPEG-CHO	CHT-mPEG-COOH
BET surface area (m^2^/g)	3.7544	3.8621	1.7418	3.6458
BJH adsorption surface area of pores (m^2^/g)	3.944	3.603	1.230	3.205
BJH desorption surface area of pores (m^2^/g)	6.3916	4.9324	1.0743	4.9547
BJH adsorption volume of pores (cm^3^/g)	0.006280	0.006969	0.003835	0.005747
BJH desorption volume of pores (cm^3^/g)	0.006723	0.007442	0.003491	0.006451
BJH adsorption average pore diameter (Å)	63.698	77.363	124.749	71.724
BJH desorption average pore diameter (Å)	42.072	60.355	129.983	52.077

The physisorption curves for all the three scaffolds corresponded to Type II isotherm thereby confirming the macroporous morphology of the scaffolds as reported under SEM analysis^55^. Assigning the porosity data for CHT as standard, the BET/BJH surface areas, pore volume, and pore diameter altered significantly with the addition of mPEG and further functionalization of mPEG from –OH through –CHO to –COOH. Since, mPEG-OH and chitosan molecules displayed only weak H-bonding interactions, the mPEG molecule didn’t interfere with the mobility of the chitosan chains. In fact, the mPEG-OH chains might have acted as plasticizer within the chitosan chains allowing increased mobility of the chains leading to formation of more porous structure^56^. In contrast, mPEG-CHO demonstrated strong interactions with chitosan molecule via –NH_2_…CHO interactions forming an imine bond. This interaction is equivalent to crosslinking and augmented the crosslinking effect of glutaraldehyde thereby causing the formation of a rigid architecture and hence notably reduced porosity (average cross-sectional pore area)^57^. In the case of CHT-mPEG-COOH, the formation of amide bond between –NH_2_ and –COOH moieties of chitosan and mPEG-COOH, respectively, led to the formation of a slightly strained structure. Therefore, the porosity of CHT-mPEG-COOH was in range or similar to that of CHT.

Coming to the hysteresis loops observed in the isotherms; all four scaffolds showed open hysteresis loops with possibility of closing at very low pressures. According to the IUPAC, this can be associated with “the swelling of a non-rigid porous structure” which conforms to the highly resilient nature of the polymeric scaffolds^58^. Except CHT-mPEG-CHO which displayed a H4 hysteresis loop, the rest of the scaffold showed H3 hysteresis loop which as per the IUPAC can be defined as “aggregates of plate-like particles giving rise to slit-shaped pores”. This was true in the case of CHT, CHT-mPEG-OH, and CHT-mPEG-COOH wherein slit-like non-circular pores were observed (see SEM analysis). In the case of CHT-mPEG-CHO, Type H4 hysteresis loop was observed which was defined by the IUPAC as “associated with narrow slit-like pore”. This appeared to be true for CHT-mPEG-CHO, wherein the comparatively more crosslinked CHT scaffold produced congested architecture formed by narrow pores.

This was further substantiated by the image analysis data wherein the % roundity of CHT-mPEG-CHO was the highest among all the cryosponges. It is well known that a circle has the least area among its corresponding concentric shapes and hence CHT-mPEG-CHO showed narrowest pores and least surface area. Although the units of surface area obtained from image analysis were different from that of porositometric analysis, the relative pore area among the scaffolds was superimposable in both the analysis with CHT-mPEG-CHO and CHT-mPEG showing the lowest and the highest surface area values, respectively. This further confirms that validity and applicability of our image processing paradigm.

### Texture analysis of CHT/mPEG cryosponges

**Figure 7:**
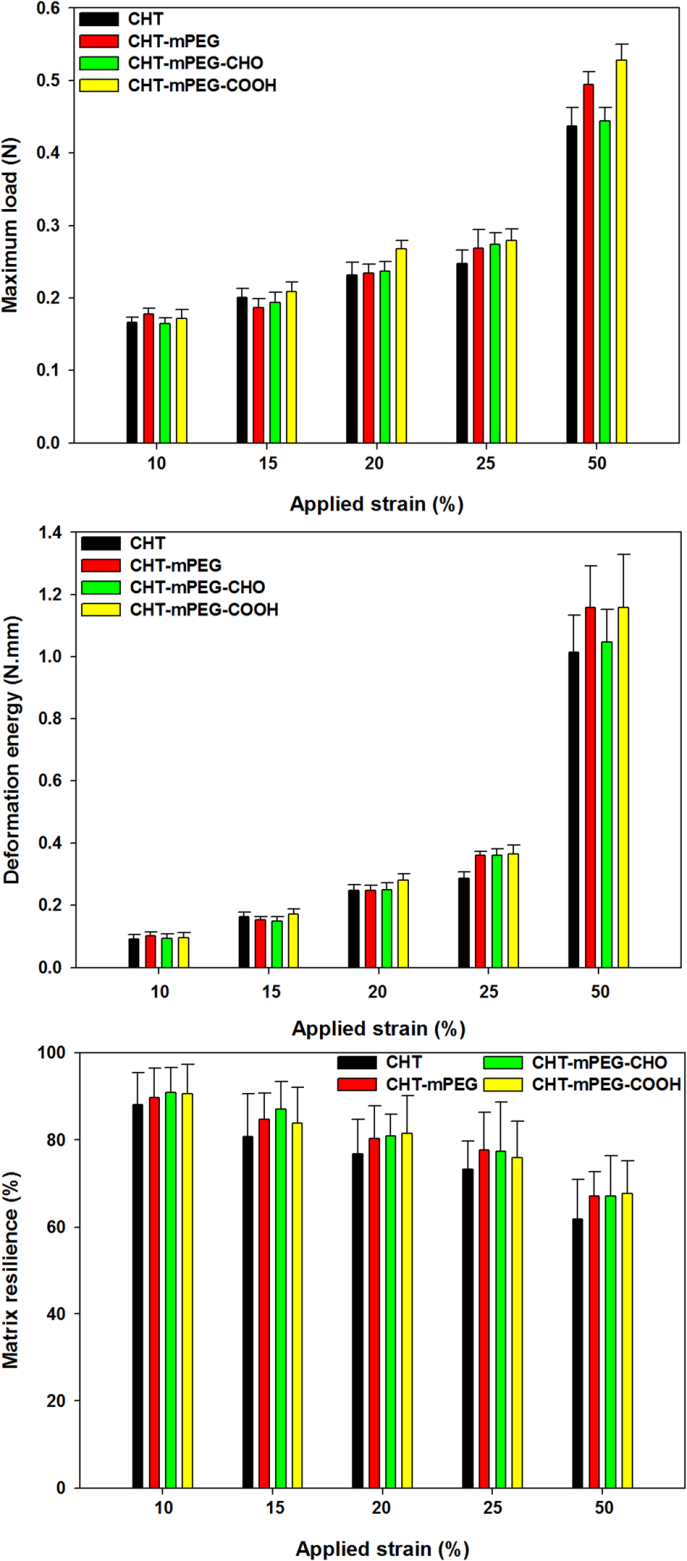
Physicomechanical properties of various CHT/mPEG cryosponge formulations under partial applied strain values of 10–50% (data represent mean ± std. dev. from 3 independent samples).

For the physicomechanical profiling of our chitosan/mPEG based scaffolds, the textural analysis was conducted on “fully” hydrated samples after thawing and washing with distilled water. The scaffolds were very robust and easy to handle with steel forceps. However, to avoid crushing the scaffolds and to prevent the loss of water while handling, flat-end plastic forceps were used to lift and place these scaffolds on the aluminium stage. The scaffolds were cut using a razor blade in their hydrated state. For texture analysis, compressive strain was applied at 10, 15, 20, 25 and 50% with identical test and post-test speeds and data was analysed to obtain maximum strength, deformation energy, and % matrix resilience (Fig. 7)^59^.

In general, the blend scaffolds displayed better mechanical properties than the CHT scaffold with slightly higher mechanical strength and resilience. The higher resilience can be attributed to the plasticization of CHT chains by mPEG and hence increasing the mobility of the chains. The higher matrix strength can be due to the higher solid content in blend scaffolds as compared to CHT alone scaffold. It is evident from the bar charts (Fig. 7) that the mechanical properties of CHT and blend scaffolds, and among blend scaffolds were not significantly different (*p *value > 0.05) except for maximum load at 50% strain in CHT-mPEG and CHT-mPEG-COOH when compared with CHT and CHT-mPEG-CHO scaffolds (*p *value < 0.05). The equivalent matrix strength, deformation energy, and resilience at all applied strain values implied that functionalization of mPEG had no evident influence on the macro-mechanical properties of the CHT-mPEG blend scaffolds. Another explanation for non-determination of the subtle mechanical transformations in the blend scaffolds could be the role played by glutaraldehyde crosslinking wherein the strong crosslinking reaction overshadowed the mechanical changes thereby making them undetectable^60^. It was hereby proposed that more sensitive techniques such as rheological mechanical analysis are required to elucidate the mechanical impact of functionalized mPEG’s interactions with chitosan in fluid state. Further details are provided under rheological analysis.

### Rheomechanical analysis using oscillation frequency sweep

**Figure 8:**
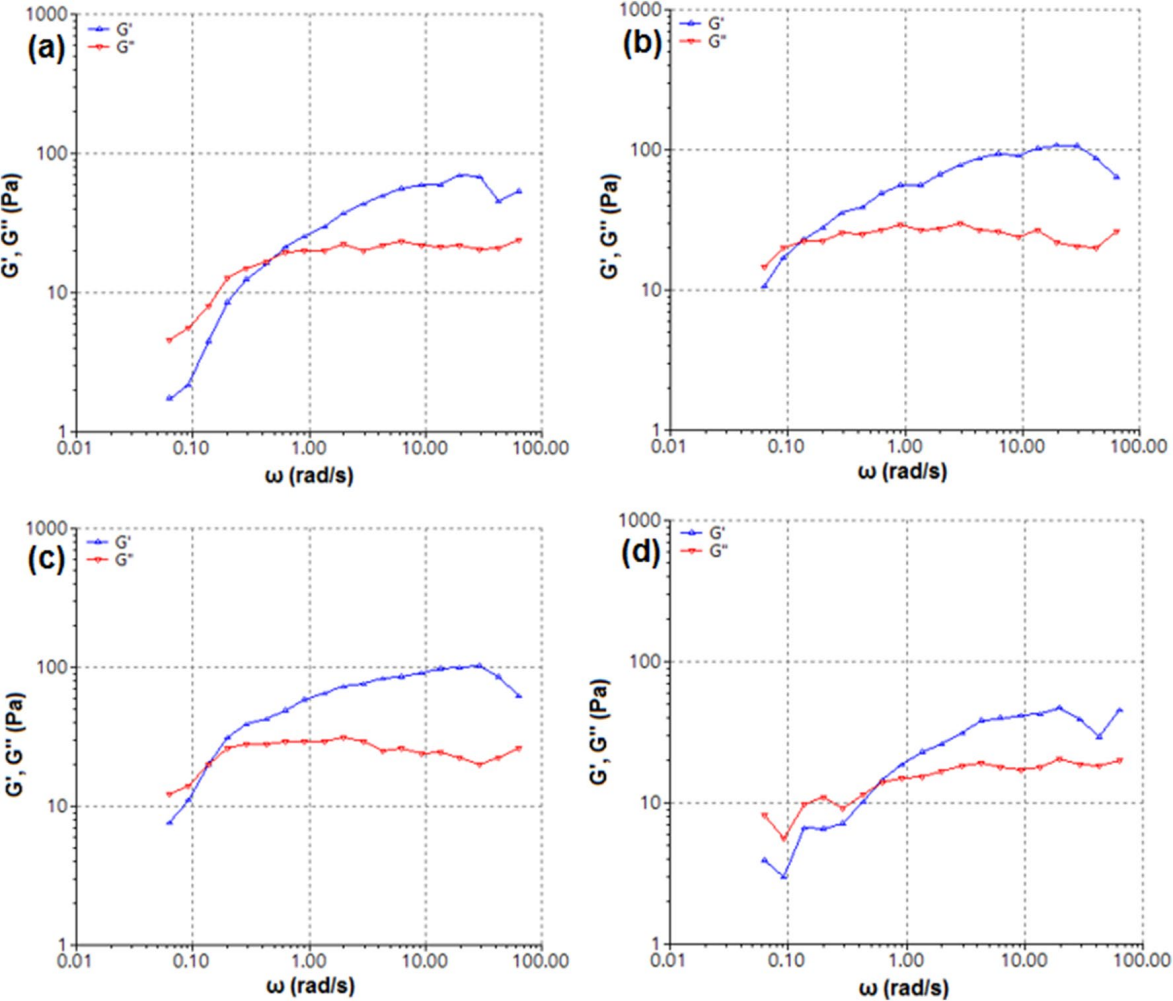
Oscillation frequency sweep (@2.0 Pa) rheograms depicting G′, G″ versus angular frequency for (**a**) CHT; (**b**) CHT-mPEG; (**c**) CHT-mPEG-CHO; and (**d**) CHT-mPEG-COOH blend solutions.

The oscillation frequency sweep curves for non-crosslinked CHT and CHT-mPEG blend solution are provided in Fig. 8. The G′, G″ vs angular frequency curve for CHT showed G″ > G′ at lower frequency ranges, a crossover at medium frequency ranges, followed by G′ > G″ at higher frequency ranges (Fig. 8a). For the blend solutions, it was theorized that with an increase in polymer concentration, rigidity, and gelation—the G′ = G″ crossover shows a horizontal shift towards lower angular frequency and a vertical shift. As expected, with the addition of mPEG to CHT, the total polymer content of the solution doubled (CHT:mPEG::1:1) leading to a significant horizontal shift of the G′ = G″ crossover (Fig. 8b, Table 5). However, this was accompanied by a slight upward vertical shift as the plasticizing function compensated the increased polymer content and provided the flexibility and mobility to the CHT chains. In the case of CHT-mPEG-COOH, the G′ = G″ crossover further shifted horizontally as a decrease in the mobility of PEG chains occurred due to –NH_2_…COOH interaction (Fig. 8d). The vertical shift, although not significant, was still upwards which can be attributed to the higher strength of the polymer composite along with the psuedo branch-like mPEG chains on the CHT chains. Finally, for CHT-mPEG-CHO, the G′ = G″ crossover occurred at the lowest frequency values due to the formation of most rigid structure among the blend formulations (Fig. 8c). The formation of relatively rigid network in CHT-mPEG-CHO was further evident from the increase in the modulus value to 22.38 Pa which was the highest among the tested formulations confirming the formation of –NH_2_…CHO cross-links as explained under FTIR and DSC discussion.

**Table 5:** Rheological parameters obtained from oscillation frequency sweep tests conducted on CHT/mPEG blend solutions.

Sample	Angular frequency at G′ = G″ crossover (rad/s)	G value at G′ = G″ crossover (Pa)
CHT	0.5067	18.24
CHT-mPEG	0.2555	17.89
CHT-mPEG-CHO	0.1301	22.38
CHT-mPEG-COOH	0.1401	20.31

### Matrix hydration and degradation analysis

To assess the response of lyophilized scaffolds towards neuronal aqueous medium (pH 7.4), the scaffold was tested for their ability to hold water (% water holding capacity; WHC), wet weight (% matrix hydration; MH), and physical degradation (PD). WHC referred to the maximum water a scaffold can hold within the matrix network as well as in the matrix pores and effectively is the aqueous medium present in the scaffold at a given time (before draining out the aqueous medium)^61^. MH was calculated by draining out the excess scaffold surface water as well as the water within the pores by absorbing the water onto a filter paper until an equilibrium weight is reached. MH should not be confused with matrix swelling as the scaffolds used were prepared by lyophilisation in their fully hydrated state and therefore showed no increase in size^62^. This approach is proposed as the most suitable method to prevent the extensive swelling of biopolymers in vivo such as for neural tissue engineering applications^63^.

CHT scaffolds showed significantly better hydration profiles at all time points (*p* value < 0.05) as compared to the blend scaffolds except for % MH in CHT-mPEG-CHO on Day 1 (p-value = 0.066). It is worth noting that the blend scaffolds showed insignificant variation in hydration among themselves (*p* value > 0.05). The lowered WHC and MH of blend scaffolds in general were due to the plasticization effect of mPEG on the intermolecular interaction of CHT chains. Decreased intermolecular interactions among the polymer chains can disrupt the H-bong making the chains more mobile and hence decreasing the water holding capacity^64–66^. Another reason for reduced WHC and MH in blend scaffolds could be the reduced availability of NH_2_ groups for swelling in under basic conditions owing to their interaction with functional groups. Among the blend formulations, CHT-mPEG-CHO showed best %WHC and %MH profiles. This can be attributed to the least plasticization function of mPEG-CHO due to interaction with NH_2_ functionality of CHT and hence reducing the interchain mobility. Additionally, such interaction appears to form a “pseudo-branched” polymer increasing the water holding capacity of the scaffold. Furthermore, as mPEG-CHO provided some degree of crosslinking among other mPEG derivatives, the strength of the CHT chain increased comparatively leading to an increase in the capacity of the scaffold to hold more water^25^.

**Figure 9:**
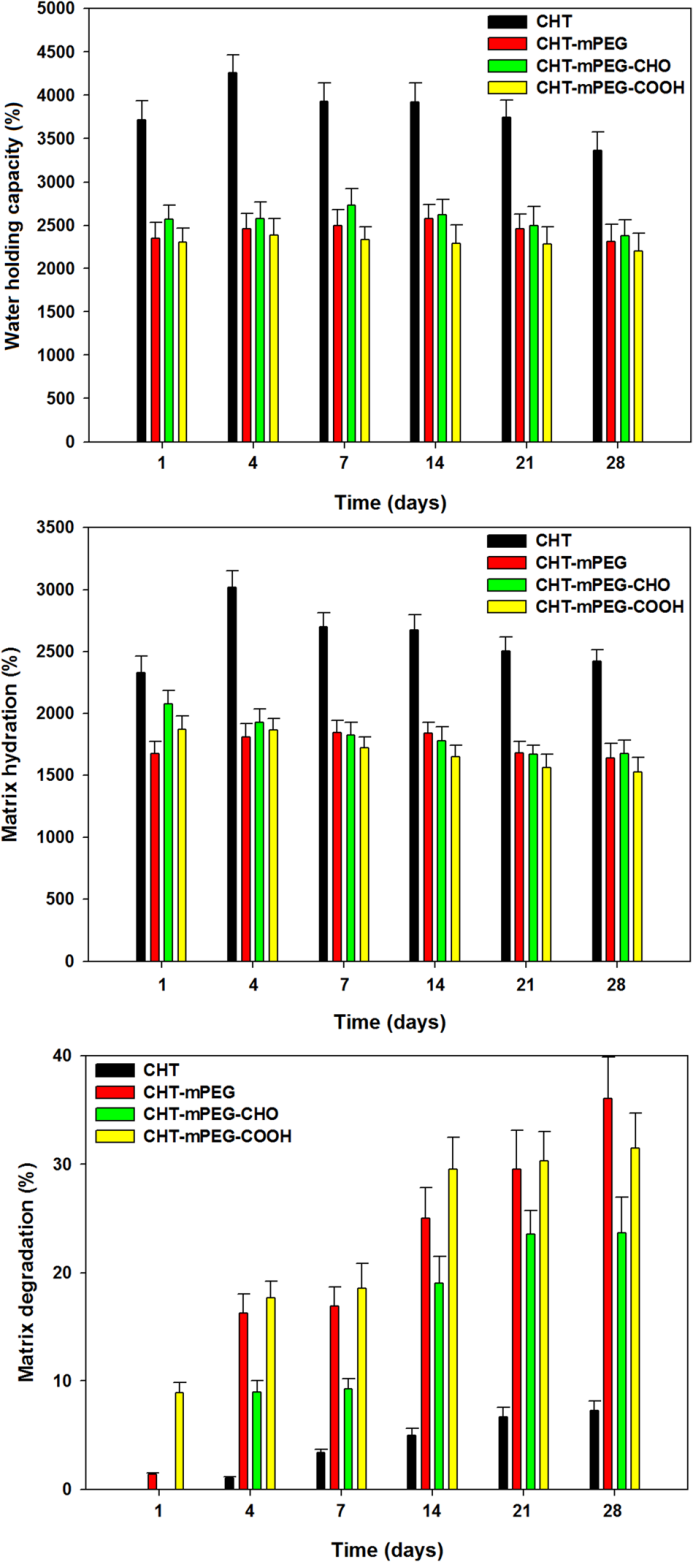
Bar charts depicting hydration and degradation profiles of various cryosponges (data represent mean ± std. dev. from 3 independent samples).

Physical degradation referred to as the degradation of the scaffold in aqueous medium and represented the ability of the scaffold to hold-together its matrix. The physical degradation data in Fig. 9 showed that CHT degraded negligibly as compared to the blend scaffolds at all time points (*p* value < 0.05). The increased degradation of the blend scaffolds can be ascribed to the mPEG assisted penetration of water into the CHT network (due to hydrophilicity of mPEG) as well as due to the bulk and surface degradation of mPEG segments. As the mPEG chains were not crosslinked as compared to CHT, they underwent faster degradation. This was further evident from the degradation profile of blend scaffolds wherein the scaffold containing free mPEG chains (CHT-mPEG) degraded faster than the scaffolds containing conjugated mPEG chains (CHT-mPEG-CHO and CHT-mPEG-COOH). The CHT-mPEG-CHO scaffold underwent slowest degradation among the blend formulations (*p* value < 0.05 at all time points except day 21) owing to the strongest conjugation of mPEG-CHO with CHT as explained under rheology and FTIR.

### Bioactive release from CHT/mPEG cryosponges

**Figure 10:**
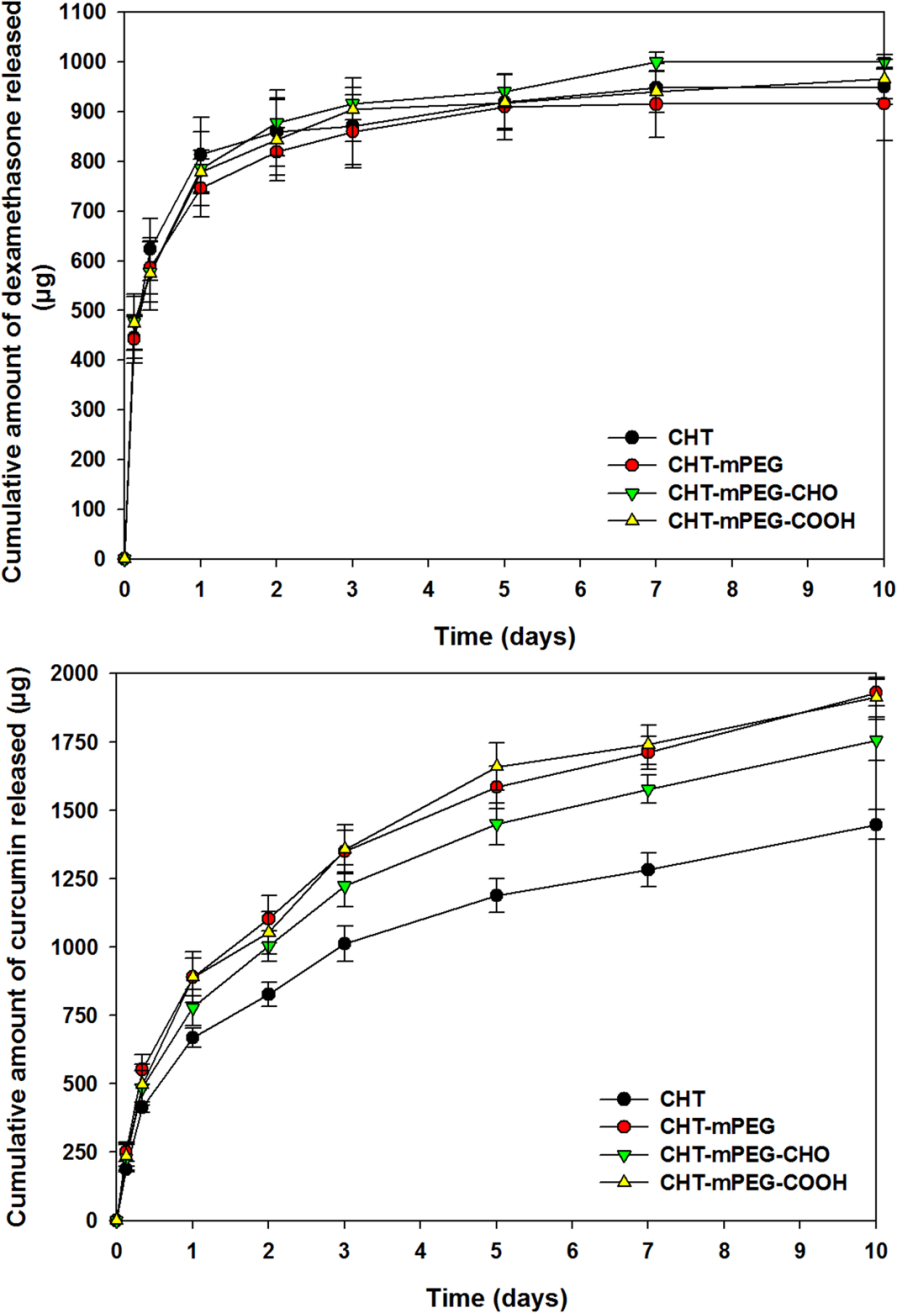
Drug release profiles of various cryosponge formulations over 10 days (data represent mean ± std. dev. from 3 independent samples).

The drug release profiles for the CHT and CHT-mPEG blends are shown in Fig. 10. The dexamethasone salt release from the scaffolds didn’t vary much among the formulations and followed a rapid release curve with 70–80% drug released in first 24 h and ≈ 90% drug released within 3 days. This can be attributed to the highly macroporous behaviour of the scaffolds as well as the hydrophilic nature of the dexamethasone disodium phosphate incorporated in the scaffolds. This was in line with previously reported release of highly water soluble dexamethasone disodium phosphate from crosslinked gelatin scaffolds^67^. However, the release profiles for the hydrophobic bioactive, curcumin, were very different from that of dexamethasone and closely followed the results obtained from matrix hydration data discussed above. As expected, the blend scaffolds displayed faster release than CHT-only scaffolds. The hydrophilicity (increased water accessibility) as well as the plasticization effect of mPEG derivatives on CHT chains immensely contributed to the increased release in the blend scaffolds. Furthermore, among the blend scaffolds, CHT-mPEG-CHO scaffolds displayed slowest release due to the formation of a crosslinked-conjugated system between -NH_2_ of chitosan and –CHO leading to formation of a more rigid network. The release data highlights the importance of faster dexamethasone release and slower curcumin release and may have simultaneous release applications as a neurotherapeutic and neuroprotective paradigm, respectively^68^.

### Elucidation of the molecular interactions using atomistic simulations

**Figure 11:**
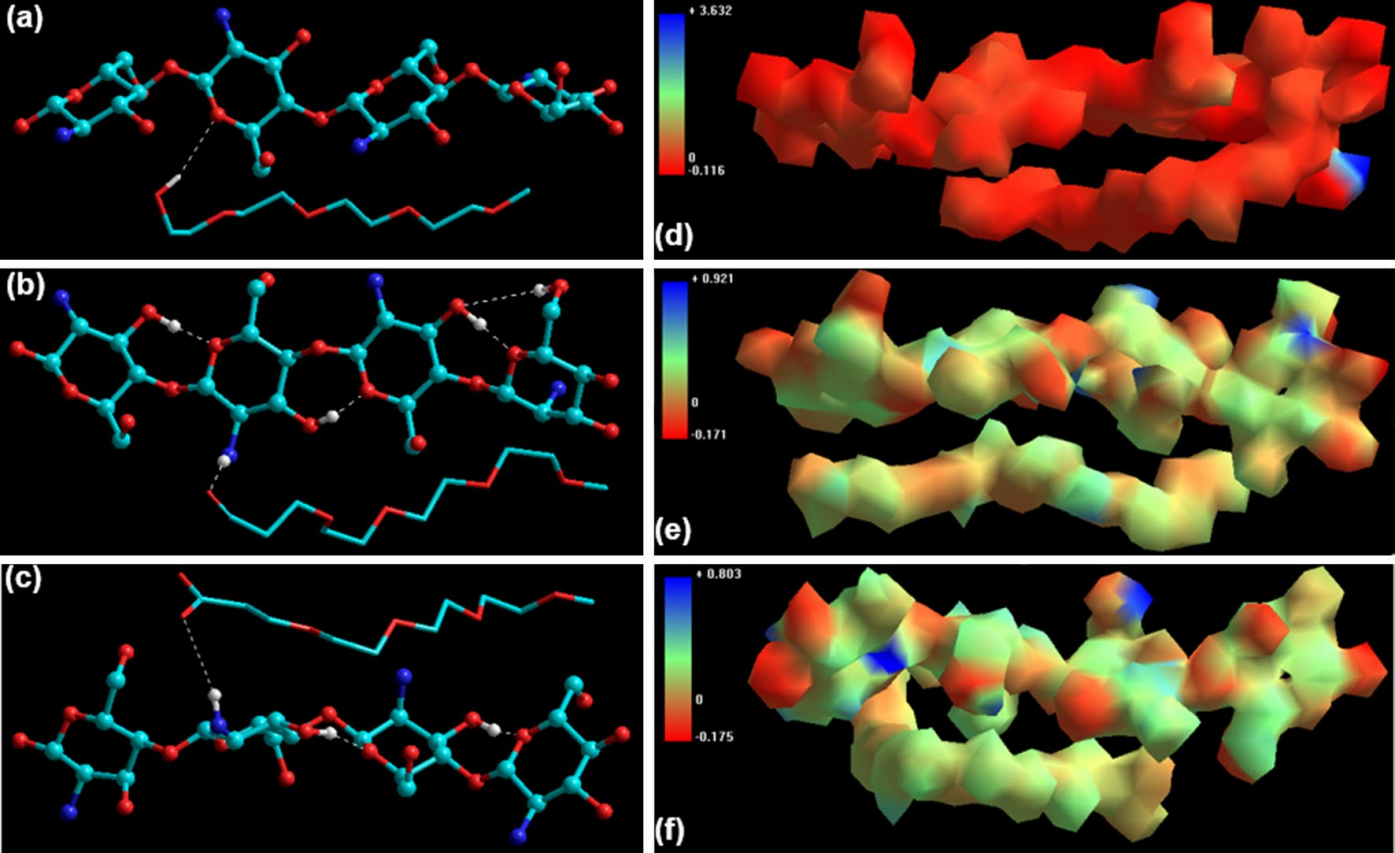
Visualization of geometrical preferences of (**a**) CHT-mPEG; (**b**) CHT-mPEG-CHO; and (**c**) CHT-mPEG-COOH molecular complexes after molecular simulation in vacuum (Color codes: C (cyan), O (red), N (blue) and H (white)) and 3D-mapped isosurface plot representing the electrostatic potential for (**d**) CHT-mPEG; (**e**) CHT-mPEG-CHO; and (**f**) CHT-mPEG-COOH molecular complexes.

Molecular mechanics simulations were carried out to confirm two major aspects of CHT/mPEG cryosponge formation: (1) the hydrogen bonding or molecular complexation between the chitosan chain and the mPEG derivatives; and (2) whether the CHT-mPEG cryosponges form an energetically stable molecular complex (Fig. 11). It was hypothesized that the underlying chemical interactions between the constituent molecular components can lead to conformational changes responsible for physicomechanical modulations in the blends.

Following observations were made with reference to CHT-mPEG molecular blends:

- The overall geometric stabilization of CHT-mPEG blends (CHT-mPEG, CHT-mPEG-CHO, and CHT-mPEG-COOH) was accompanied by steric energy minimization of − 19.374, − 4.375, and − 7.806 kcal/mol, respectively (Table 6). This confirmed the compatibility and stability of the CHT-mPEG molecular blends. The energy stabilization in the case of CHT-mPEG-CHO and CHT-mPEG-COOH was significantly lower as compared to CHT-mPEG which can be attributed to –NH_2_…CHO and –NH_2_…COOH H-bonding as shown in Fig. 11. Such H-bonding leads to steric torsion, stretching, and bending which further cause the formation of relatively strained and destabilized CHT-mPEG-CHO and CHT-mPEG-COOH molecular structures.
- The most crucial finding of this molecular simulation study was the electrostatic interactions within the CHT-mPEG-CHO and CHT-mPEG-COOH blends. The highly stabilized electrostatic interaction energy values of − 51.372 and − 57.353 kcal/mol can be attributed to the intramolecular bonding within the chitosan chain such as N–H…C–O–C and OH…OH in close vicinity of the mPEG chains as well as due to the intermolecular interaction between the functional groups (CHT–N–H…OHC and CHT–N–H…HOOC) of the component molecules, respectively. Most importantly, the molecular graphs demonstrated significant shift of electrostatic mapping from − 0.116/+ 3.632 for CHT-mPEG to − 0.171/+ 0.921 (CHT-mPEG-CHO) and − 0.175/+ 0.803 (CHT-mPEG-COOH). The introduction of –CHO and –COOH functionalities in the blend transitioned the “majorly positive” CHT-mPEG to much electrostatically balanced molecular complexes due to the involvement and consumption of –N–H groups in molecular complex formation.

**Table 6:** Computational energy attributes calculated for the simulated CHT-mPEG blend system in a molecular mechanics’ force field setup.

Energy Attributes (kcal/mol)	CHT	mPEG	CHT-mPEG	mPEG-CHO	CHT-mPEG-CHO	mPEG-COOH	CHT-mPEG-COOH
*V*_*∑*_^a^	15.222	16.918	12.766	15.411	26.258	19.117	26.533
ΔE^b^	–	–	− 19.374	–	− 4.375	–	− 7.806
*V*_*b*_^c^	1.288	0.109	1.375	0.146	2.149	0.149	2.541
*V*_*θ*_^d^	7.393	0.528	8.303	0.770	11.285	0.772	13.060
*V*_*φ*_^e^	10.106	15.404	17.563	13.079	71.832	16.882	74.425
*V*_*ij*_^f^	6.726	0.876	− 0.059	1.415	− 7.637	1.313	− 6.141
*V*_*el*_^g^	− 10.292	0.000	− 10.417	0.000	− 51.372	0.000	− 57.353

^a^Minimized global energy for an optimized structure; ^b^ΔE_interaction_ = E(Host.Guest) − E(Host) − E(Guest); ^c^bond stretching contributions; ^d^bond angle contributions; ^e^torsional contribution arising from deviations from optimum dihedral angles; ^f^van der Waals interactions; ^**g**^electrostatic energy.

### Determination of preliminary neuronal cell compatibility

**Figure 12:**
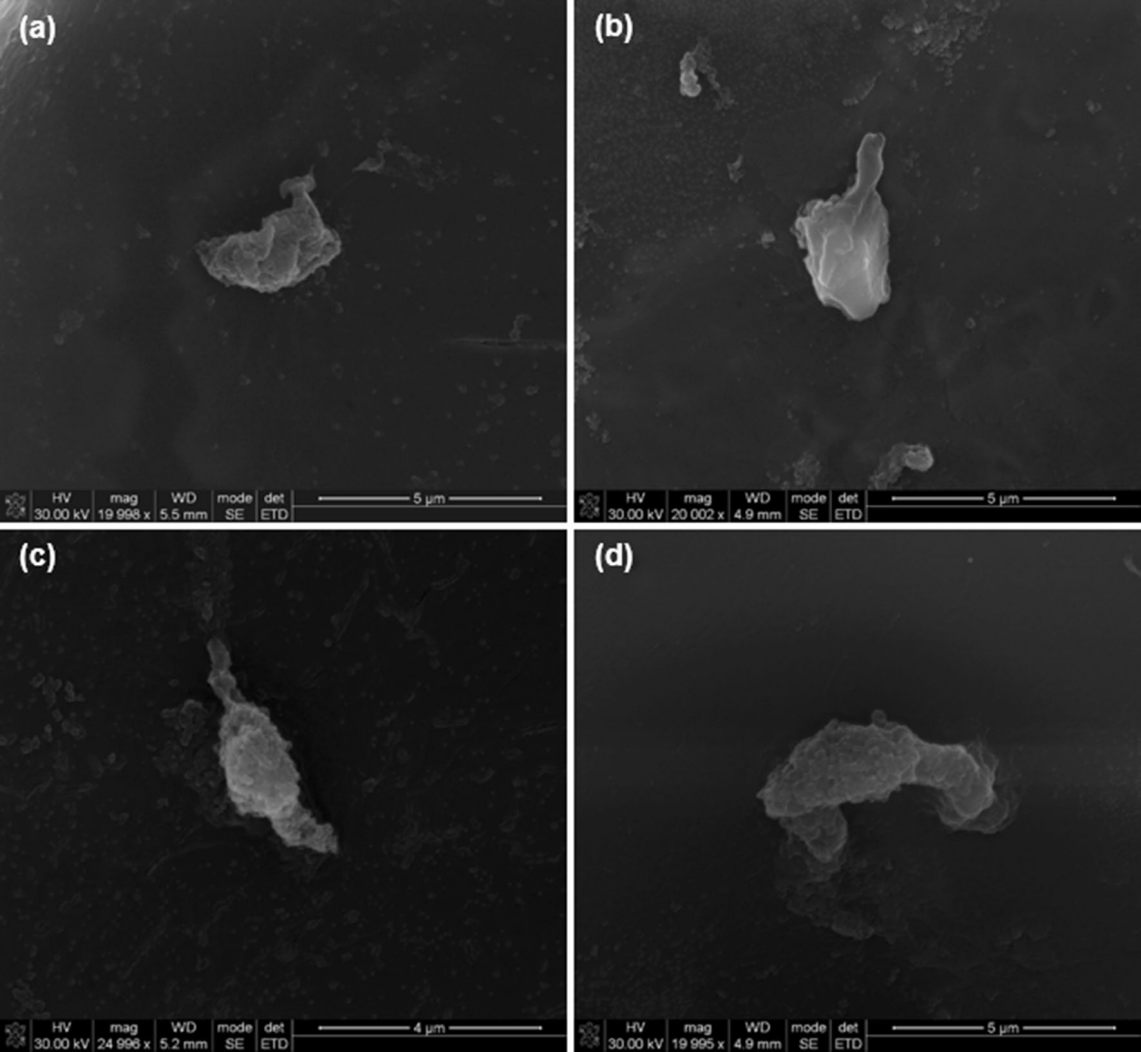
Representation of the PC12 attachment on cryosponge surfaces showing potential cell attachment (**a**) CHT; (**b**) CHT-mPEG; (**c**) CHT-mPEG-CHO; and (**d**) CHT-mPEG-COOH.

The preliminary cell study indicated that the CHT-mPEG cryosponges were capable of efficiently supporting the growth of PC12 cells (derived from pheochromocytoma of the rat adrenal medulla) over a period of 72 h. This confirmed the potential neurocompatibility of the cryosponges as platform systems (Fig. 12). The morphological assessment clearly reflected the effect of chemical functionalization in CHT/mPEG cryosponges wherein –CHO and –COOH functionalized mPEG derivatives demonstrated notable neuronal attachment and adhesion of the cryosponges endorsing the “chemo-biological outreach” proposition^69,70^.

## Conclusion

In this study, novel mPEG-blend-chitosan scaffolds were successfully prepared using crosslinking-cryogelation method under sub-zero conditions and were characterised for various physicochemical, physicomechanical, and morphological properties. The morphology of the cryosponges presented surface features such as fringe thread-like structures (CHT), fringe thread-like structures with bulbs (CHT-mPEG), rough shallow ridges (CHT-mPEG-CHO), and elephant-skin-type ridges (CHT-mPEG-COOH). The cryosponges further presented unique morphological structures such as hemispherical, pebble-like structures (CHT-mPEG), curved quartz crystal-like or crystal-flower-like structures (CHT-mPEG-CHO), and grouped, congealed, steep-sided canyon-like structures (CHT-mPEG-COOH) confirming the role of minimal functionalization on the morphological modification of biomaterial-based scaffolds. Such minimal functionalization also affected the rheomechanical properties of the scaffolds and hence played a significant role in matrix degradation and drug release from the scaffolds. The molecular simulation studies depicted the involvement of various functional groups involved in matrix blending and polymer interaction. In addition, the cryosponges provided cell adhesion properties as described by preliminary PC12 studies. Further in vitro cell studies, in vivo biocompatibility, and biodegradation studies needs to be undertaken to further prove the role of distinctive morphology in the biomedical performance of implanted scaffolds for regenerative medicine such as for neurotrauma and wound healing applications.

## Materials and methods

### Materials

Chitosan (CHT) low molecular weight (50–150 kDa; η = 370.6 mPas for a 1%^w^/_v_ solution in 1%^v^/_v_ acetic acid solution at 25 °C), PEG methyl ether (mPEG; 5 kDa), mPEG acetic acid (mPEG-COOH; 5 kDa), glutaraldehyde, curcumin, and dexamethasone disodium phosphate were obtained from Sigma–Aldrich, St. Louise, MO, USA. All other reagents used were of analytical grade and were used as received. mPEG-aldehyde (mPEG-CHO) was synthesized by the oxidation of mPEG with anhydrous dimethylsulfoxide/acetic anhydride [Supplementary material Scheme S1]^71^.

### Preparation of chitosan/mPEG cryosponges

Low molecular weight chitosan (1.5 g) was dissolved in 90 mL of aqueous acetic acid solution (0.5%^v^/_v_). After complete dissolution of chitosan, 1.5 g of mPEG derivative was added to the above chitosan solution and allowed to dissolve for 30 min. To fabricate the tubular cryosponges, 1 mL of 2%^v^/_v_ glutaraldehyde was added to 9 mL of CHT/mPEG solution, vigorously stirred for 10 s, poured in polypropylene syringe moulds and immediately frozen at − 20 °C for 12 h. After 12 h, the cryosponges were thawed at room temperature, washed twice in sequence with 2% glycine solution and double distilled water, transferred to polypropylene petri dishes, and were frozen overnight at − 80 °C. The frozen cryosponges were then lyophilized (FreeZone 2.5, Labconco, Kansas City, MS, USA) at 25mtorr for 24 h at − 42 °C. For preparation of CHT-only cryosponge, no mPEG derivative was added to the CHT solution and same procedure was followed as explained above.

### Morphological analysis and image processing

For morphological analysis, the scaffold matrices were sputter coated with carbon and/or chromium and photomicrographs was captured at various magnifications using FEI Nova Nanolab 600 SEM, FEI, Hillsboro, Oregon, USA (30 kV; 2 K image resolution (2048 × 1768). The micrographs so obtained were extensively analyzed and quantified using DiameterJ and ND plugins created for ImageJ and FIJI (image processing softwares). As defined and described by the inventors, DiameterJ plugin is a “diameter measurement tool” and was created “using existing algorithms for centerline determination, Euclidean distance transforms and a novel pixel transformation technique”. A unique algorithm combining the segmentation tool of DiameterJ and the Image Threshold Adjustment function of ImageJ was developed for the processing of SEM micrographs. The processed images were then analysed for pore area and fractional roundity^52,53^.

### Porositometric analysis of the cryosponges

Porositometric analysis was performed on the cryosponges using a porosity analyser (Micromeritics ASAP 2020, Norcross, GA, USA). The scaffold samples were cut with a razor blade and accurately weighed (100 mg) before adding to the samples tube. Given the high and multilevel porosity of the scaffolds, the scaffolds were degassed for 22 h at 40 °C. After degassing, the sample was transferred to the analysis port and a data report incorporating surface area, pore volume, and pore size related to adsorption and desorption isotherms. Both BJH and BET computations were evaluated. BET theory measures the specific surface area of materials while BJH theory is a pore size distribution determination method. The linear isotherm plots obtained were compared with the guidelines as prescribed by the IUPAC^54,55^. Further details of the method and equipment settings can be found in previous reports published elsewhere^72^.

### Polymeric structural variation analysis

To determine the molecular-structural transformations within the cryosponges, Attenuated Total Reflectance-FTIR (ATR-FTIR) was performed on the native components (chitosan and mPEG derivatives) and the final scaffolds using a MIRTGS detector (PerkinElmer Spectrum 100, Llantrisant, Wales, UK). Samples were scanned (64 scans/spectrum) within 650–4000 cm^−1^ wavelength at a resolution of 4 cm^−1^.

### Exothermic and endothermic mapping of the grafted polymers

Differential Scanning Calorimetry (DSC) analyses were performed on the native components (chitosan and mPEG derivatives) and the final scaffolds (Mettler Toledo, DSC1, STAR^e^ System (Schwerzenback, Switzerland) Accurately weighed samples (5 mg ± 0.1 mg) were placed into an aluminum sample holder and experimental runs were performed by heating the samples at a heating rate of 5 °C/min from 10 to 325 °C under a constant flow of N_2_ gas.

### Physicomechanical characterization of the cryosponges

#### Textural macroanalysis

The micromechanical properties of the scaffold may directly influence the ability of the axons to regenerate, proliferate and penetrate within the scaffold matrix. Textural profile analysis was therefore conducted at a micro-scale employing a Texture Analyzer (TA.XT*plus* Stable Microsystems, Surrey, UK) fitted with a 5 kg load cell. The scaffold matrices were cut into cylinders using a scalpel (10 mm diameter; 10 mm length) and were compressed under various strain values between 10 and 50%. The scaffold matrices were placed on an aluminium stage and were compressed using a flat probe. Serial Force–Time/Distance profiles were generated for various formulations using the parameters detailed in Table 7. Mechanical computations with reference to maximum load, deformation energy, rigidity gradient and % matrix resilience were carried out^59^ in triplicate (n = 3) and the data was analysed using a two-tailed student t-test (Supplementary material Figure S1).

**Table 7:** Textural parameter settings employed for physicomechanical property analysis of the cryosponges.

Test parameters	Settings	
	Matrix deformation	Matrix resilience
Pre-test speed	1 mm/s	1 mm/s
Test speed	1 mm/s	0.5 mm/s
Post-test speed	5 mm/s	0.5 mm/s
Compressive strain (%)	10, 15, 20, 25, and 50	10, 15, 20, 25, and 50
Sensitivity of trigger force	0.04903 N	0.04903 N

#### Rheological measurements of constituent polymer blend solutions

Rheometric analysis was conducted for CHT-alone (yield value ≈ 0.1670 Pa) and CHT/mPEG blend mixtures (yield value ≈ 0.2406 Pa) to assess the mechanical transitions inherent within the polymeric solution phase. A cone-and-plate Haake MARS (Modular Advanced Rheometer System) rheometer (Thermo Electron Corporation, Karlsruhe, Germany) with cone diameter of 35 mm, cone angle = 1° (sensor C35/1°) Ti, A-factor = 8.905e+04 Pa/Nm, M-factor = 57.01 (1/s)/(rad/s), inertia = 1.721e−06 kg m^2^, damping = 30.00, thermal expansion coefficient: 1.100 µm/°C, compliance = 0.003157 rad/Nm, and a cone/plate gap of 0.51 mm. The temperature was maintained at 20 °C using a MARS II Universal Temperature Controller and a solvent trap was employed to prevent evaporation of the sample. Oscillation frequency sweeps were conducted over a frequency range of 10–0.01 Hz at a constant stress value of 2.0 Pa which was within the linear viscoelastic region of 0.1–7.0 Pa (stress sweep analysis conducted at 0.01 Hz). The variation and behaviour of elastic and loss moduli with reference to increasing frequency (angular frequency—ω) was studied and reported as mechanical characteristics. Further details of the method and equipment settings can be found in author’s previous report published elsewhere^73^.

### Assessment of matrix hydration and degradation

For the determination of matrix hydration and degradation behaviour of the cryosponges, samples weighing 20 mg were added to 10 ml of simulated cerebrospinal fluid. The studies were performed for 28 days and the samples were taken out on days 1, 4, 7, 14, 21, and 28. The study medium was replaced every alternate day. The scaffolds holding the aqueous medium were carefully lifted with flat forceps and directly weighed to obtain the % water holding capacity (WHC; Eq. 1). To determine the matrix hydration (MH), the scaffolds were then placed on a lab tissue paper to drain the excess medium until constant weight is obtained^74^ (Eq. 2). For the determination of % matrix degradation (MD), the above samples were air dried until constant weight was obtained (Eq. 3). In case of MH and MD, the samples were essentially washed twice with distilled water and drained subsequently to remove the residual salts. The various parameters at time, t, analysed using a two-tailed student t-test.

1 $$ {\text{WHC }}\left( {\text{\% }} \right) = \left( {\frac{{{\text{W}}_{fh} - {\text{W}}_{i} }}{{{\text{W}}_{i} }}} \right) \times 100 $$

where W_i_ is the initial weight of the sample and W_fh_ is the weight of the fully hydrated sample at time t.

2 $$ {\text{MH }}\left( {\text{\% }} \right) = \left( {\frac{{{\text{W}}_{hs} - {\text{W}}_{i} }}{{{\text{W}}_{i} }}} \right) \times 100 $$

where W_i_ is the initial weight of the sample and W_hs_ is the weight of the hydrated sample after draining the aqueous medium at time t.

3 $$ {\text{MD }}\left( {\text{\% }} \right) = \left( {\frac{{{\text{W}}_{{\text{i}}} - {\text{W}}_{{\text{t}}} }}{{{\text{W}}_{{\text{i}}} }}} \right) \times 100 $$

where W_i_ is the initial weight of the sample and W_t_ is the weight of the sample at time t.

### In vitro bioactive release studies

The bioactive released from the cryosponges was quantified over time by an in vitro release assay. The polymer blend solutions were loaded with a known quantity of the bioactive and then underwent cryogelation as explained under “Structural variations analysis using FTIR” section. Scaffolds incorporating 1000 μg and 2000 μg equivalent of dexamethasone and curcumin, respectively, were placed in sealed glass jars containing 10 mL of simulated cerebrospinal fluid at 37 °C. At designated time points, 500µL aliquots of the release medium were sampled and the same quantity of fresh simulated cerebrospinal fluid was added. The cumulative amount of bioactive released as a function of time was determined by UV spectrophotometric analysis at 240 nm and 425 nm for dexamethasone and curcumin, respectively (Varian Cary 50 Conc UV–Vis Spectrophotometer, Agilent Technologies, Santa Clara, CA, USA).

### Preliminary PC12 cell culture studies

PC12 cells were employed to ascertain the potential of the scaffolds as platform systems. The protocol employed herein has been published elsewhere by authors. The cell were maintained in DMEM supplemented with DES, FBS and P/S/AB solution (complete culture medium) in a humid 5% CO_2_ atmosphere at 37 °C. The scaffold samples were sterilized under UV light for 12 h before overnight incubation in 400μL of complete culture medium. The cells were then seeded (2 × 10^4^ cells/well) onto the scaffolds and incubated for 72 h (n = 3). For morphological assessment, the cells were primed with 50 ng/mL Nerve Growth Factor in low serum media (DMEM supplemented with 1%^v^/_v_ DES) before seeding. The samples were then dip washed with PBS and lyophilized for SEM viewing ^11^.

### In silico molecular simulation and analysis

Molecular mechanics simulations and electrostatic mapping were performed to elucidate the molecular interactions within the cryosponges (ChemLite 30, Granville, FL, USA). The molecules were disposed in parallel and then MM+ force field was applied using the Polak-Rebiere gradient as default settings^75^. Component energy values such as total steric energy, bond energy, angle energy, torsional strains, van der Waals forces, and electrostatic interactions were then computed and compared.

## Supplementary Information

Supplementary Information.
