# Supplementary material for: Macroporous chitosan/methoxypoly(ethylene glycol) based cryosponges with unique morphology for tissue engineering applications
Source: Sci Rep. 2021 Feb 4;11:3104. doi: 10.1038/s41598-021-82484-x (PMC7862315; doi:10.1038/s41598-021-82484-x)
Supplement: Supplementary file 1 — Supplementary Information. [file 41598_2021_82484_MOESM1_ESM.pdf]

Macroporous chitosan/methoxypoly(ethylene glycol) based cryosponges with unique morphology for tissue engineering applications

Pradeep Kumar, Viness Pillay and Yahya E. Choonara *

Wits Advanced Drug Delivery Platform Research Unit, Department of Pharmacy and Pharmacology, School of Therapeutic Sciences, Faculty of Health Sciences, University of the Witwatersrand, Johannesburg, 7 York Road, Parktown 2193, South Africa

*** Corresponding Author:**
Professor Yahya E. Choonara
Tel: +27-11-717-2052
Fax: +27-11-642-4355
Email: yahya.choonara@wits.ac.za

SUPPLEMENTARY MATERIAL

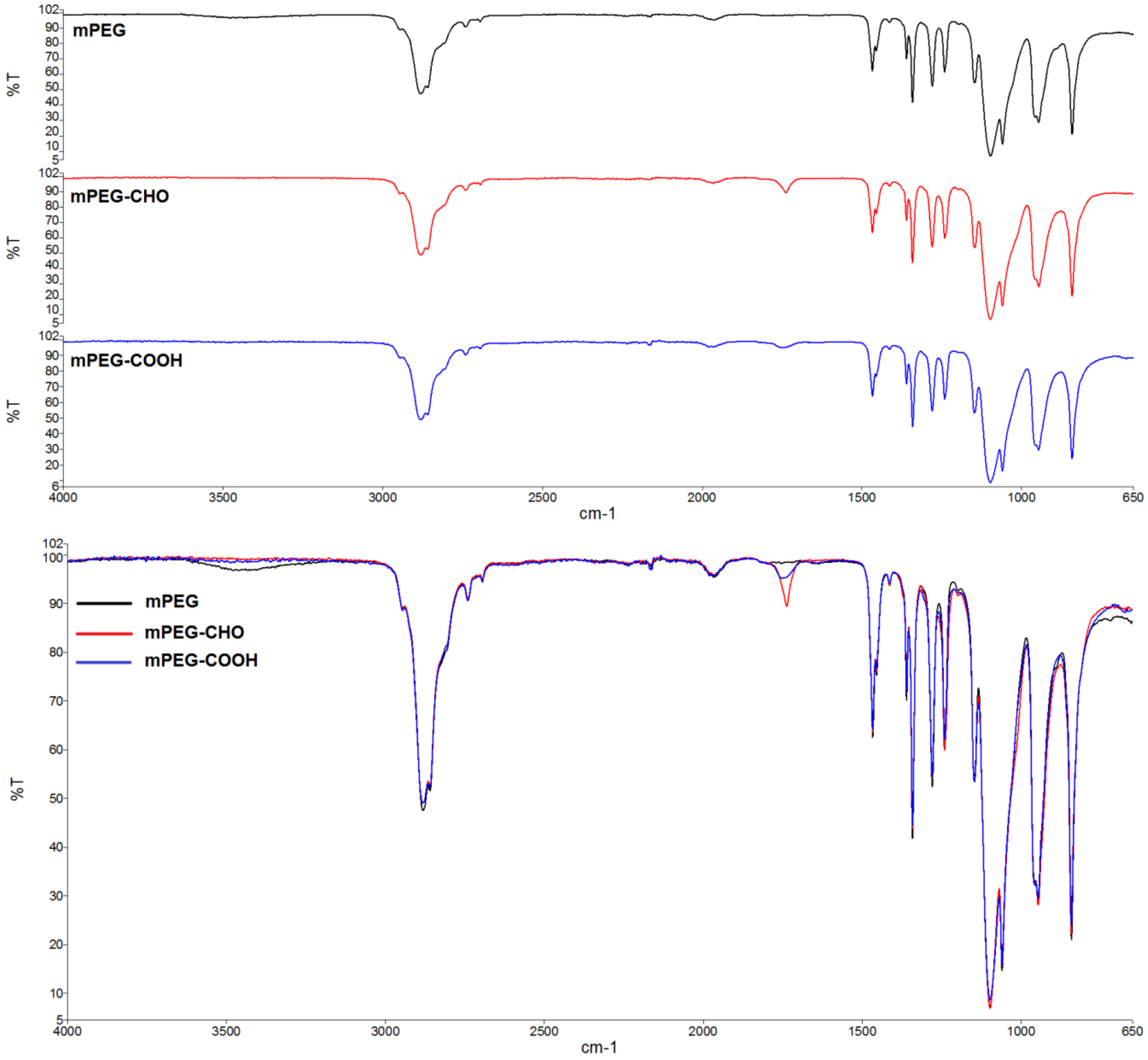

Figure S1: FTIR spectra of pristine mPEG derivatives.

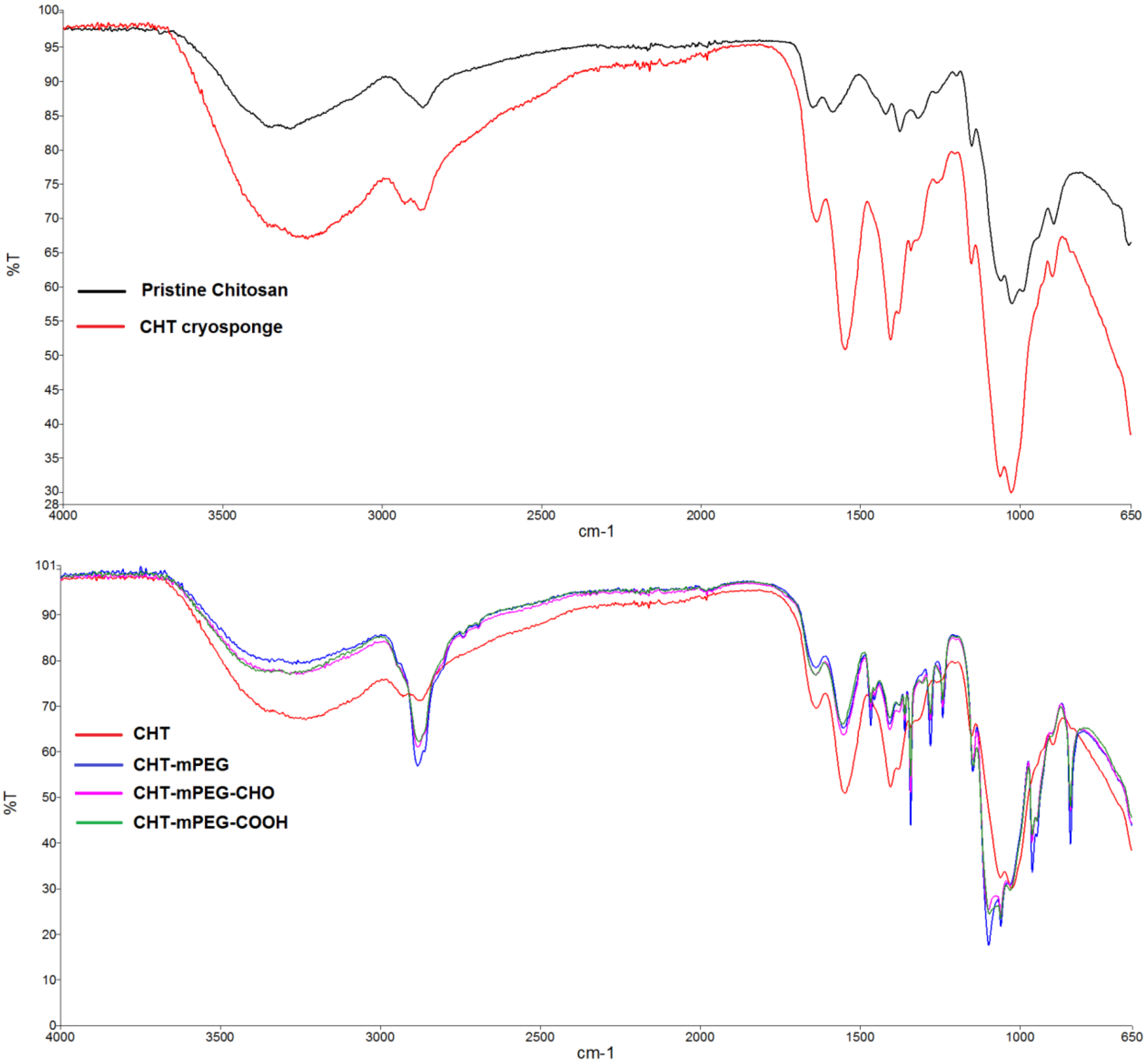

Figure S2: FTIR spectra confirming the formation of CHT and CHT/mPEG cryosponges.

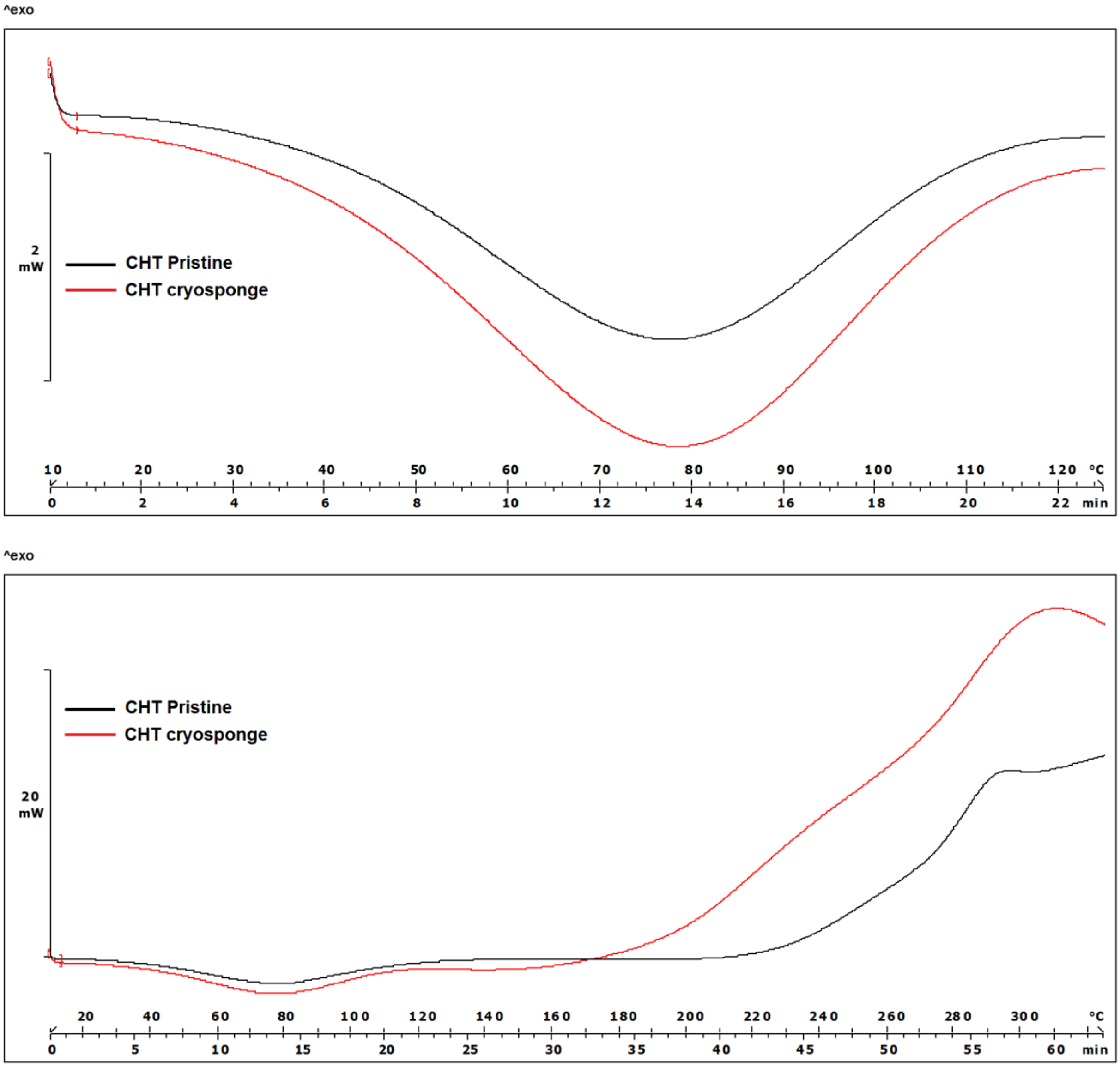

Figure S3: DSC thermograms of pristine chitosan and the CHT cryosponge over various temperature ranges.

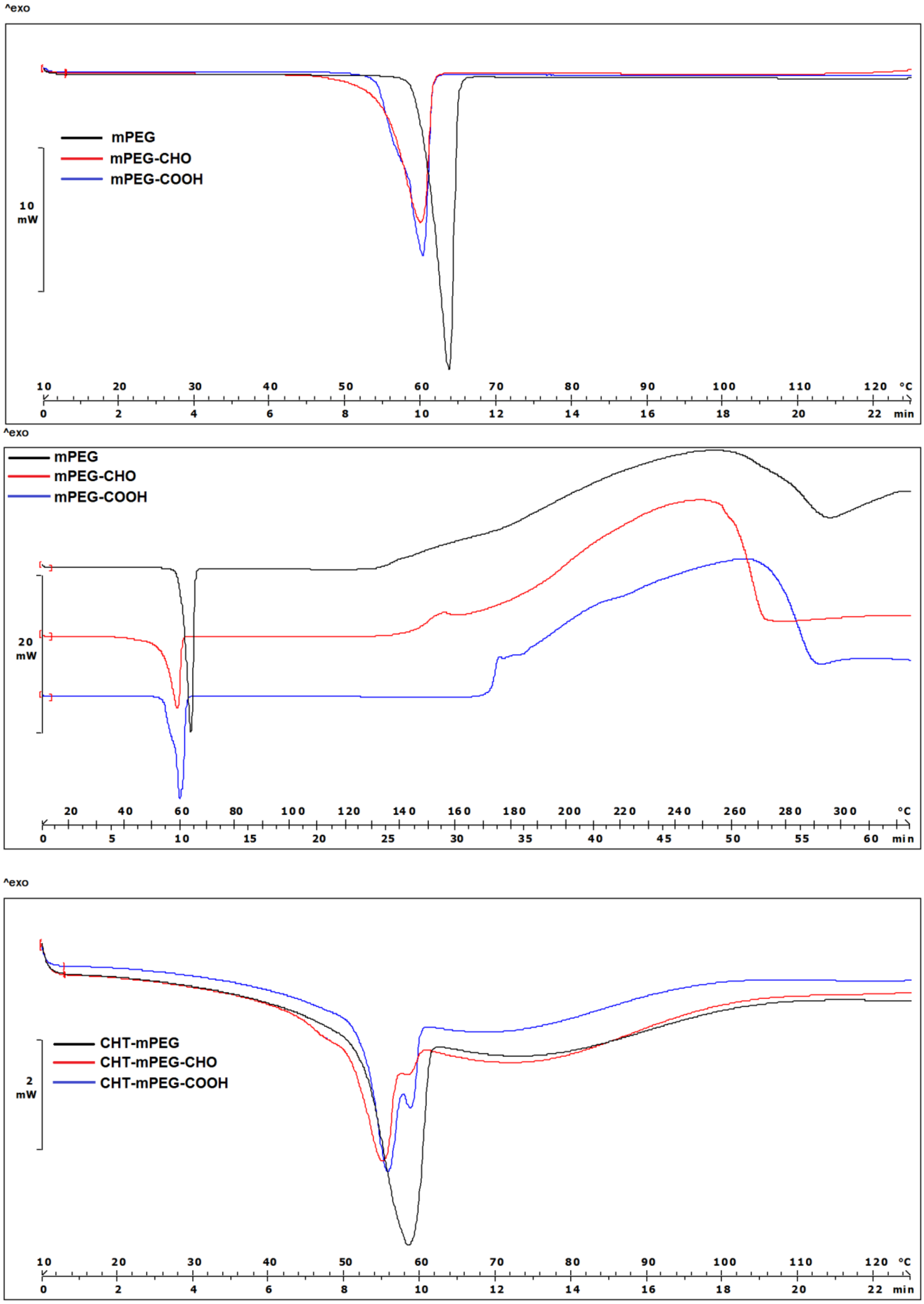

Figure S4: DSC thermograms of the pristine polymers and CHT/mPEG cryosponges over various temperature ranges.

S1. Synthesis of mPEG-aldehyde (mPEG-CHO)

mPEG-aldehyde (mPEG-CHO) was prepared by the oxidation of mPEG with anhydrous dimethylsulfoxide/acetic anhydride. Acetic anhydride (10.2mL) was added to mPEG (20g) in 60mL anhydrous dimethylsulfoxide containing 4mL chloroform under a N_2 atmosphere and the mixture was stirred for 14 hours at 20°C. The reaction mixture was then poured into 400mL anhydrous diethyl ether. The precipitate was filtered with a paper filter and re-precipitated twice from chloroform solution with diethyl ether and dried in a desiccator.

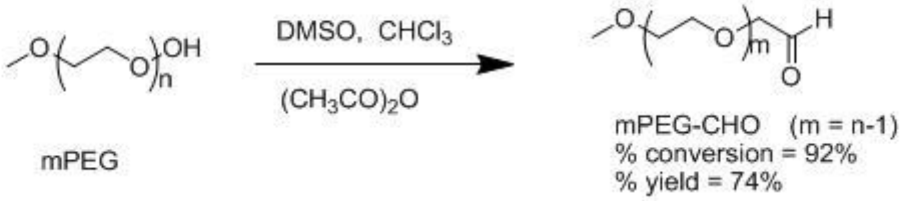

Scheme S1: Synthesis of mPEG-aldehyde

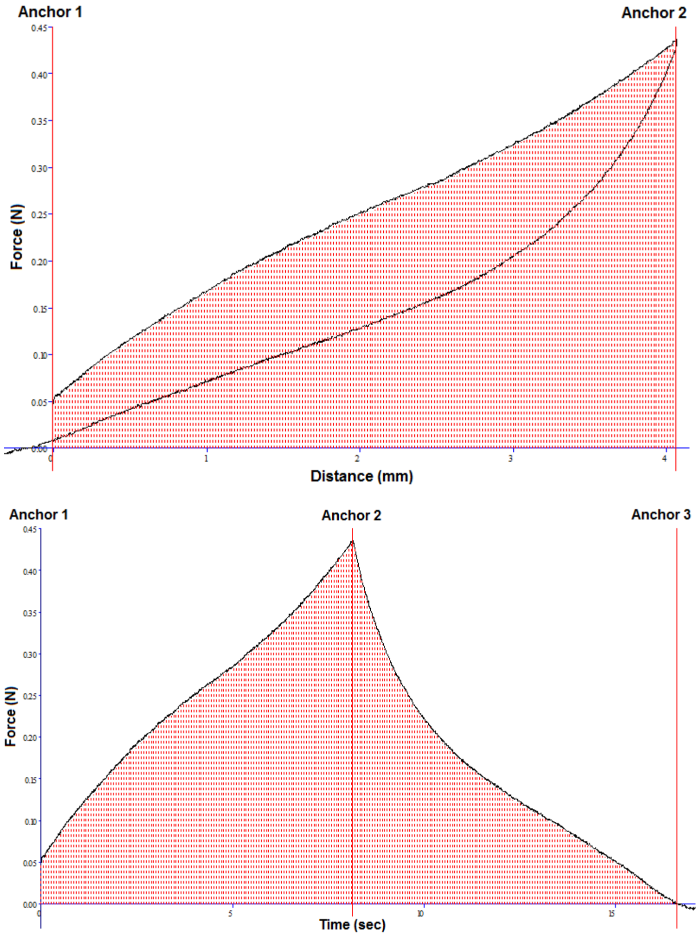

Figure S5: Typical force-distance profile representing the compression mechanical testing performed on the spinomimetic scaffolds. a) The area between anchors 1 and 2 (shaded part of the figure) represents the deformation energy while the gradient corresponds to rigidity gradient. The highest point in the curve corresponds to the maximum load experienced by the sample under the applied strain. b) The ratio of area 2 (between anchor 2 and 3) and area 1 (between area 1 and 2) represents matrix resilience of the scaffold.
